# Multi-Window Temporal Context for ECG-Based Sleep Apnea Detection Under Limited Apnea-Label Availability

**DOI:** 10.3390/diagnostics16182939

**Published:** 2026-09-11

**Authors:** Semin Ryu, Jeonghwan Koh, In cheol Jeong

**Affiliations:** 1 Department of Artificial Intelligence Convergence, Hallym University, Chuncheon 24252, Republic of Korea; jeonghwan.koh@hallym.ac.kr; 2 Cerebrovascular Disease Research Center, Hallym University, Chuncheon 24252, Republic of Korea; 3 Department of Population Health Science and Policy, Icahn School of Medicine, Mount Sinai, New York, NY 10029, USA

**Keywords:** sleep apnea detection, single-lead ECG, temporal context, data-efficient learning, label-efficient learning, multi-window modeling

## Abstract

**Background/Objectives**: Supervised electrocardiography-based sleep apnea detection often depends on dense segment-level annotations, creating substantial expert burden. To reduce this dependence, we investigated whether adjacent temporal context could improve classification when ground-truth apnea labels are limited. **Methods**: We systematically evaluated five context lengths and seven apnea-label retention levels across 35 experimental configurations using participant-grouped five-fold cross-validation with three training repetitions. **Results**: The results demonstrated that using multi-window temporal context consistently improved classification performance across the evaluated apnea-label retention range. All multi-window configurations achieved higher mean F1-scores than the corresponding single-window baseline, with the best-performing multi-window setting at each retention level providing gains of 7.18–12.23 percentage points. Context length showed a clear overall effect on performance, whereas no systematic interaction was observed between context length and apnea-label retention. Targeted repeated-center and random-shuffle control experiments further supported a contribution from temporally adjacent ECG information rather than sequence-length expansion, repeated target presentation, or temporally distant same-recording context. **Conclusions**: Overall, leveraging adjacent temporal context provides a practical strategy for improving ECG-based apnea classification and may be particularly useful for developing screening models when dense expert-provided apnea annotations are limited.

## 1. Introduction

Obstructive sleep apnea (OSA) is a highly prevalent sleep-related breathing disorder characterized by recurrent cessations or reductions in respiratory airflow during sleep, and it is associated with increased cardiovascular and metabolic morbidity [1,2]. Although polysomnography (PSG), or technically adequate home sleep apnea testing in uncomplicated adults, remains central to the diagnostic pathway, conventional in-laboratory PSG is constrained by its resource requirements, operational costs, and associated patient burden [1,2]. To reduce this burden, recent research has increasingly focused on scalable, cost-effective screening approaches based on single-lead electrocardiography (ECG), which is well suited for integration into wireless wearable and home-monitoring devices [3,4,5].

A fundamental challenge in training robust deep learning architectures for single-lead ECG analysis is their reliance on large, meticulously annotated segment-level datasets [6,7]. For model development, manual epoch-by-epoch labeling of apneic events from multi-hour overnight recordings requires substantial expert effort and introduces inter-rater variability [8,9]. Although our prior work systematically benchmarked the role of morphology-preserving time-domain data augmentation as a baseline strategy for mitigating performance loss across varying label retention ratios (r=5% to 100%) [10], that analysis was inherently limited to isolated, independent 60-s ECG segments and did not account for the rich, time-varying cardiorespiratory dependencies that extend across adjacent temporal windows.

Crucially, the cardiorespiratory manifestations associated with sleep apnea are not necessarily confined to an isolated 60-s epoch; rather, they may involve sequential, cyclic fluctuations in cardiac rhythms and respiration-modulated waveform components that span several minutes. Consequently, requiring a model to infer apneic patterns from an isolated 60-s window, particularly under conditions of limited label availability, may restrict its ability to resolve ambiguous segment-level dynamics. To address this limitation, this study introduces a multi-window evaluation framework that leverages adjacent temporal context to stabilize and improve apnea detection under limited supervision.

To provide a systematic empirical evaluation, we constructed a 35-case experimental matrix spanning seven apnea-label retention levels (r={5, 10, 20, 40, 60, 80, 100}%) and five context window configurations (k={1, 3, 5, 7, 9}), where *k* denotes the number of contiguous 1-min ECG segments used for block-level inference. All experiments were conducted using a deterministic, participant-grouped five-fold cross-validation protocol that prevents recordings from the same participant from being shared between the training and test partitions, thereby supporting evaluation on unseen participants [10,11]. Our objective was not to maximize absolute detection performance or compete with state-of-the-art architectures, but rather to isolate and quantify the effect of adjacent temporal context (*k*) on performance across different levels of apnea-label retention (*r*).

The primary contributions of this research are threefold:

1. We propose a multi-window temporal context framework that integrates neighboring ECG segments across multiple context lengths to address the limitations of single-segment apnea classification under limited supervision.
2. We present a comprehensive 35-case empirical evaluation crossing multiple apnea-label retention levels with context window configurations, providing a transparent and reproducible benchmark for data-efficient biosignal modeling.
3. We demonstrate that the benefit of adjacent temporal context is broadly maintained across apnea-label retention levels and use targeted control experiments to distinguish the contribution of true neighboring ECG information from effects attributable to sequence-length expansion, repeated target presentation, or temporally distant same-recording context.

## 2. Related Studies

### 2.1. ECG-Based Sleep Apnea Detection and Localized Window Bias

Automated detection of sleep apnea from single-lead ECG recordings has evolved significantly from classical heart rate variability (HRV)-based feature extraction methods to end-to-end deep learning approaches [12,13]. Recent studies have explored lightweight convolutional models, hybrid convolutional-recurrent architectures, and transformer-based sequence encoders for single-lead ECG-based apnea detection [4,14,15,16]. These architectures have demonstrated strong performance in capturing local P–QRS–T complex morphology and structural patterns within short ECG segments.

However, because the PhysioNet Apnea-ECG benchmark dataset provides annotations at a native 1-min epoch resolution [17], many existing approaches formulate apnea detection using isolated 60-s analysis windows without explicitly incorporating neighboring temporal context. Although these models often perform well under favorable training conditions, treating consecutive temporal blocks as independent and identically distributed observations may introduce a localized window bias. In practice, OSA involves multi-minute cardiorespiratory cycles during which HRV and respiration-modulated waveform components evolve gradually across adjacent epochs [11]. Restricting a classifier to a single isolated minute may limit its effective receptive field, leaving potentially informative temporal transitions underutilized and increasing prediction ambiguity at segment boundaries, particularly under conditions of limited apnea-label availability. For example, epochs occurring near transitions between apneic and normal breathing may contain ambiguous within-epoch morphology, whereas the immediately preceding and following ECG segments can provide contextual evidence regarding the evolving cardiorespiratory state.

### 2.2. Sequence-Level Modeling vs. Explicit Multi-Window Context

To address the limitations of isolated epoch processing, recent studies have explored sequence-level approaches that ingest multi-hour or full-night ECG recordings using recurrent architectures or deep state-space models [18]. These continuous-processing frameworks aim to propagate global temporal representations across extended overnight recordings. However, full-night sequence modeling increases sequence length, optimization complexity, and computational resource requirements, and may require careful regularization, particularly when labeled training data are limited. In contrast, our framework avoids the computational and optimization overhead associated with full-night sequence processing by employing an explicit, localized multi-window context (k∈{1, 3, 5, 7, 9}). This approach bounds the temporal scope to a fixed set of neighboring segments, providing local contextual information that may stabilize classification without increasing the parameter budget or compromising optimization under conditions of limited apnea-label availability. Accordingly, the objective of the present study is not to establish superiority over full-night sequence models, but rather to isolate the contribution of explicitly defined local temporal context under controlled architecture and apnea-label retention settings.

### 2.3. Label-Efficient Configurations and Biosignal Augmentation

To address the substantial financial and logistical costs associated with clinical annotation, research in label-efficient biosignal processing has increasingly focused on semi-supervised learning, self-supervised pre-training, and generative data synthesis [9,19,20]. Contrastive temporal encoders and transferable ECG representation models have demonstrated the ability to learn robust feature representations from large unlabeled or source-domain ECG corpora [20,21]. In parallel, a wide range of deep generative architectures has been investigated for synthetic ECG generation [22], while diffusion-based frameworks [23] and state-space augmented transformers [24] have been proposed to address class imbalance in ECG datasets.

Despite their methodological sophistication, advanced generative and self-supervised approaches often introduce substantial computational overhead, require extensive hyperparameter tuning, and may be susceptible to issues such as mode collapse or distributional shifts relative to real physiological data [25]. Conversely, our framework deliberately isolates the data-efficient contribution of temporal context expansion within a purely supervised setting, establishing a transparent baseline that seeks to maximize the utility of limited annotated data without relying on external modalities or generative models for fully synthetic ECG generation [10]. To maintain this focus, semi-supervised and self-supervised methods were not included as direct baselines; such approaches represent distinct strategies for label efficiency and would introduce additional pre-training objectives and training protocols beyond the scope of the present comparison.

### 2.4. Multi-Window and Contextual Configurations in Broader Time-Series Tasks

Although the systematic use of multi-window temporal context has received limited attention in ECG-based sleep apnea detection, leveraging adjacent temporal frames is a well-established strategy in other time-series and biosignal applications. In automatic speech recognition, contiguous acoustic context frames and look-ahead windows are routinely used to resolve instantaneous phonetic ambiguities [26]. Similarly, in human activity recognition and mobile sensor-based locomotion tracking, rolling multi-window sequences derived from multi-axial accelerometers and gyroscopes are widely employed to capture transitions between activities, such as the shift from standing to walking [27,28]. In multivariate sensor data mining, aggregating neighboring sliding windows is a common strategy for suppressing local noise and identifying long-term anomalies [29,30]. Building on this concept, the present study introduces a systematic 35-case experimental framework (r×k) to evaluate how temporal block context may compensate for limited manual segment-level supervision in ECG-based apnea classification.

## 3. Materials and Methods

### 3.1. Overview of the Evaluation Pipeline

The empirical pipeline used to evaluate the joint effects of temporal context and apnea-label retention comprises three stages: signal preprocessing, participant-grouped five-fold cross-validation, and controlled apnea-label retention combined with multi-window construction and data augmentation. Unlike conventional single-lead ECG classifiers that process each epoch independently, the proposed framework groups consecutive epochs into explicit temporal blocks to assess performance under limited apnea-label supervision. In this scheme, the central epoch provides the classification target, whereas the neighboring epochs are used only as unlabeled temporal context. The model is trained end-to-end using a compact CNN–BiLSTM backbone that incorporates bidirectional sequence modeling and attention-based pooling.

### 3.2. Signal Preprocessing and Shared Anchor Construction

Experiments were conducted using the publicly available PhysioNet Apnea-ECG benchmark database [17,31]. The database comprises continuous single-lead ECG recordings annotated at a fixed 1-min resolution for apneic and normal events [17]. The analysis included 69 recordings after excluding recording c05, as detailed in Section 3.3. All recordings were uniformly sampled at 100 Hz and segmented into non-overlapping 60-s epochs of 6000 samples each according to the ground-truth annotations [17]. Among the 33,847 annotated 60-s epochs, 74 individual windows failed quality-control criteria, comprising 41 truncated tail windows and 33 near-flat windows; no non-finite windows were identified. Following the preprocessing protocol described in our previous study [10], each recording was band-pass filtered between 0.5 and 40 Hz using a zero-phase fourth-order Butterworth filter to attenuate baseline wander, powerline interference, and high-frequency muscle artifacts while preserving the diagnostically relevant P–QRS–T frequency range. Each segment was subsequently standardized using local *z*-score normalization, and amplitudes exceeding ±4σ were clipped to reduce the influence of extreme outliers while largely preserving waveform morphology. The resulting valid 1-min segments were stored as float32 tensors with dimensions of (1,6000).

To ensure that all context-length conditions were evaluated using exactly the same target epochs, a shared anchor set was defined based on the widest temporal configuration (k=9). After excluding 552 recording-edge annotations that could not support a complete nine-epoch context, 33,295 candidate anchors remained. Of these candidates, 164 were rejected because at least one epoch within the corresponding nine-epoch context failed the validity criteria, whereas no candidate was rejected because of temporal discontinuity. An epoch was retained as an eligible anchor only when four valid preceding epochs and four valid succeeding epochs were available within the same recording and all nine constituent epochs satisfied the required 60-s temporal continuity criterion. The final shared anchor set contained 33,131 target epochs, comprising 20,208 normal and 12,923 apneic epochs, corresponding to an apnea prevalence of 39.01%. Overall, 716 of the 33,847 annotated epochs (2.12%) were excluded from the shared target-anchor set because of recording-boundary or signal-validity requirements, while no additional participant group was removed as a consequence of anchor construction. Because anchor eligibility was determined solely by recording position, signal validity, and temporal continuity rather than by the apnea/normal label, the selection procedure did not explicitly favor either class; however, we did not separately characterize the excluded epochs and therefore cannot rule out systematic differences between excluded and retained epochs. The same 33,131 target-anchor identifiers were used for all context lengths (k=1, 3, 5, 7, and 9), and the shorter context tensors were constructed as exact centered subsets of the corresponding k=9 tensors. Boundary epochs were excluded rather than padded because padding would introduce artificial temporal context and violate the requirement that all constituent windows contain observed ECG data. Although this provides a controlled comparison across context lengths, real-world recordings containing missing or interrupted segments may require alternative strategies such as masking, imputation, or variable-length context handling. Detailed quality-control and shared-anchor construction statistics are provided in Supplementary Table S1.

### 3.3. Participant-Grouped Cross-Validation Protocol

To ensure rigorous evaluation while preventing participant-level data leakage, the 69 included recordings were conservatively organized into 31 participant groups before five-fold cross-validation partitioning [32]. Under this protocol, all recordings assigned to the same participant group were kept within a single partition, ensuring that no participant group was shared across the training, validation, and testing sets. Recording c05 was excluded because c05 and c06 originate from the same original recording, with c05 beginning 80 s after c06, as documented by PhysioNet [33]. To preserve participant independence conservatively, c06 was grouped with x33 based on the participant metadata linking c05 to x33; the detailed rationale is provided in Supplementary Methods. The resulting participant groups were assigned to five outer folds containing 6–7 participant groups and 13–14 recordings each. Fold construction was optimized to balance recording counts, normal-anchor counts, apneic-anchor counts, total-anchor counts, and apnea prevalence across folds, using a fixed split seed of 42 for reproducibility. The resulting outer-fold apnea prevalence ranged from 37.81% to 40.42%, compared with 39.01% in the complete anchor set. Using participant-level apnea–hypopnea index (AHI) values derived from the original Apnea-ECG metadata, the mean AHI across folds ranged from 23.03±33.15 to 30.46±32.80 events/h, with an overall value of 26.20±27.47 events/h.

For each outer-fold evaluation, one outer fold served as the test set, the subsequent fold in cyclic order served as the validation set, and the remaining three folds were used for training. Specifically, test folds 0–4 were paired with validation folds 1, 2, 3, 4, and 0, respectively. The outer-fold assignment remained fixed across all context lengths, apnea-label retention conditions, and experimental repetitions, ensuring that paired comparisons were performed on identical participant-level partitions. Exact participant-to-record mappings, participant-level AHI values, and fold-level class and AHI distributions are provided in Supplementary Tables S2 and S3.

### 3.4. Multi-Window Context Configuration and Apnea-Label Retention Protocol

A key component of this study is the construction of a 35-case evaluation matrix that combines seven apnea-label retention ratios (r∈{5, 10, 20, 40, 60, 80, 100}%) with five context window configurations (k∈{1, 3, 5, 7, 9}). The retention ratio *r* was applied exclusively to the training partition, whereas the validation and test sets were left unchanged [10]. All normal training anchors were retained at every value of *r*, whereas only approximately r% of the real apnea-labeled training anchors were retained as supervised target samples. Accordingly, *r* represents the retention fraction of real apnea training labels and should not be interpreted as the fraction of all training labels retained. For example, under r=5%, the overall fraction of unique real training anchors retained ranged from 62.43% to 63.58% across folds because all normal anchors remained available. For r<100%, the reduction in real apnea target samples was compensated for through offline data augmentation so that the effective number of positive training samples, and therefore the effective training-set size, matched the corresponding r=100% reference within each fold. Accordingly, within the effective apnea-class training pool, approximately 95%, 90%, 80%, 60%, 40%, 20%, and 0% of the samples were offline-augmented at *r* = 5%, 10%, 20%, 40%, 60%, 80%, and 100%, respectively, with the remainder consisting of retained real apnea target samples. Exact fold-specific real and augmented sample counts are provided in Supplementary Table S4.

Let $x_{t}\in R^{1\times 6000}$ denote the target 1-min ECG segment at temporal index *t*. Rather than presenting $x_{t}$ to the network in isolation, a symmetric temporal context block, $X_{t}^{\left(k\right)}$, was constructed by aggregating *k* adjacent windows centered on *t*:

(1) $$X_{t}^{\left(k\right)}=\left[x_{t-\lfloor k/2\rfloor },\ldots ,x_{t},\ldots ,x_{t+\lfloor k/2\rfloor }\right]\in R^{k\times 1\times 6000}$$

where k∈{1, 3, 5, 7, 9} determines the context length. These configurations correspond to input durations of 1, 3, 5, 7, and 9 min, respectively. Candidate context blocks were generated with a one-epoch (60-s) sliding stride, after which only anchors satisfying the shared k=9 eligibility criteria were retained; for example, when k=3, consecutive valid blocks take the form $\left[x_{1},\;x_{2},\;x_{3}\right]$ centered on and labeled by $x_{2}$, followed by $\left[x_{2},\;x_{3},\;x_{4}\right]$ centered on $x_{3}$, and then $\left[x_{3},\;x_{4},\;x_{5}\right]$ centered on $x_{4}$. Regardless of the value of *k*, the classification target remained the central epoch, $x_{t}$. The classification task remained binary (normal versus apnea) for all values of *k*. The block $X_{t}^{\left(k\right)}$ was assigned only the ground-truth label of $x_{t}$, while the neighboring windows served only as contextual information and did not contribute additional labels. Because the shared target-anchor set had already been defined using the widest context (k=9), the blocks for k=1, 3, 5, and 7 were constructed as exact centered subsets of the corresponding k=9 blocks.

Label removal affected only whether an apnea anchor was used as a supervised central training target. An apnea epoch excluded from the supervised target set could therefore still appear as an unlabeled neighboring context epoch for another retained target anchor. Across k=3–9, apnea-labeled epochs accounted for 5.86–8.20% of neighboring window occurrences at r=5% and 8.54–10.69% at r=10%. Among apnea epochs excluded from the supervised target set, 17.37–49.93% at r=5% and 25.81–67.49% at r=10% were nevertheless re-exposed at least once as unlabeled neighboring context, depending on context length. Complete statistics for this unlabeled contextual exposure are provided in Supplementary Table S9. Accordingly, *r* represents the scarcity of supervised apnea target labels rather than the complete removal of apnea-related ECG morphology from the model input; neighboring labels themselves were neither provided to the model nor incorporated into the training loss. Three paired experimental repetitions were performed using subsampling seeds 42, 43, and 44 and training seeds 142, 143, and 144, respectively. Within each fold, repetition, and retention level, the same retained apnea-anchor subset was used across all five values of *k*, preserving paired comparisons across context lengths. Combined with the five participant-grouped folds, the 35 experimental conditions and three repetitions yielded a total of 525 primary trained models.

### 3.5. Dual-Stage Augmentation Strategy

To stabilize model optimization under conditions of limited apnea-label retention, we adopted the two-stage augmentation framework described in our previous study [10], which combines offline dataset expansion with online mini-batch perturbation. The offline stage was applied exclusively to the retained real apneic training pool when r<100%, generating morphology-preserving variants until the number of apneic samples matched that of the full-retention reference setting. Offline augmentation included temporal shifting, amplitude scaling, Gaussian noise, sinusoidal baseline perturbation, time warping, and short signal dropout.

The online stage, in contrast, was applied to every mini-batch sample as a lightweight regularization strategy [30]. It introduced additional stochastic temporal shifts, amplitude perturbations, Gaussian noise, and short masking intervals during training. For multi-window inputs, both offline and online transformations were sampled independently for each constituent 60-s window rather than being shared across the complete context block. No data augmentation was applied to the validation or test partitions. Augmentation types, transformation probabilities, and magnitude ranges were held constant across all context-length and apnea-label retention conditions whenever augmentation was applied; only the number of offline-generated apnea samples varied as a function of *r*. Detailed transformation probabilities and magnitude ranges are provided in Supplementary Table S5. The same augmentation framework was quantitatively evaluated in our previous study using spectral similarity, R-peak-aligned mean-beat morphology, QRS duration, R–R intervals, and ECG-derived respiratory variability, supporting preservation of key morphological and physiological characteristics of apnea ECG signals [10]. To provide a direct visualization under the present experimental protocol, representative pooled mean-beat comparisons for the low-retention conditions (r=5% and 10%) are additionally provided in Supplementary Figure S3.

### 3.6. Attention-Pooled CNN-BiLSTM Architecture

**Figure 1:**
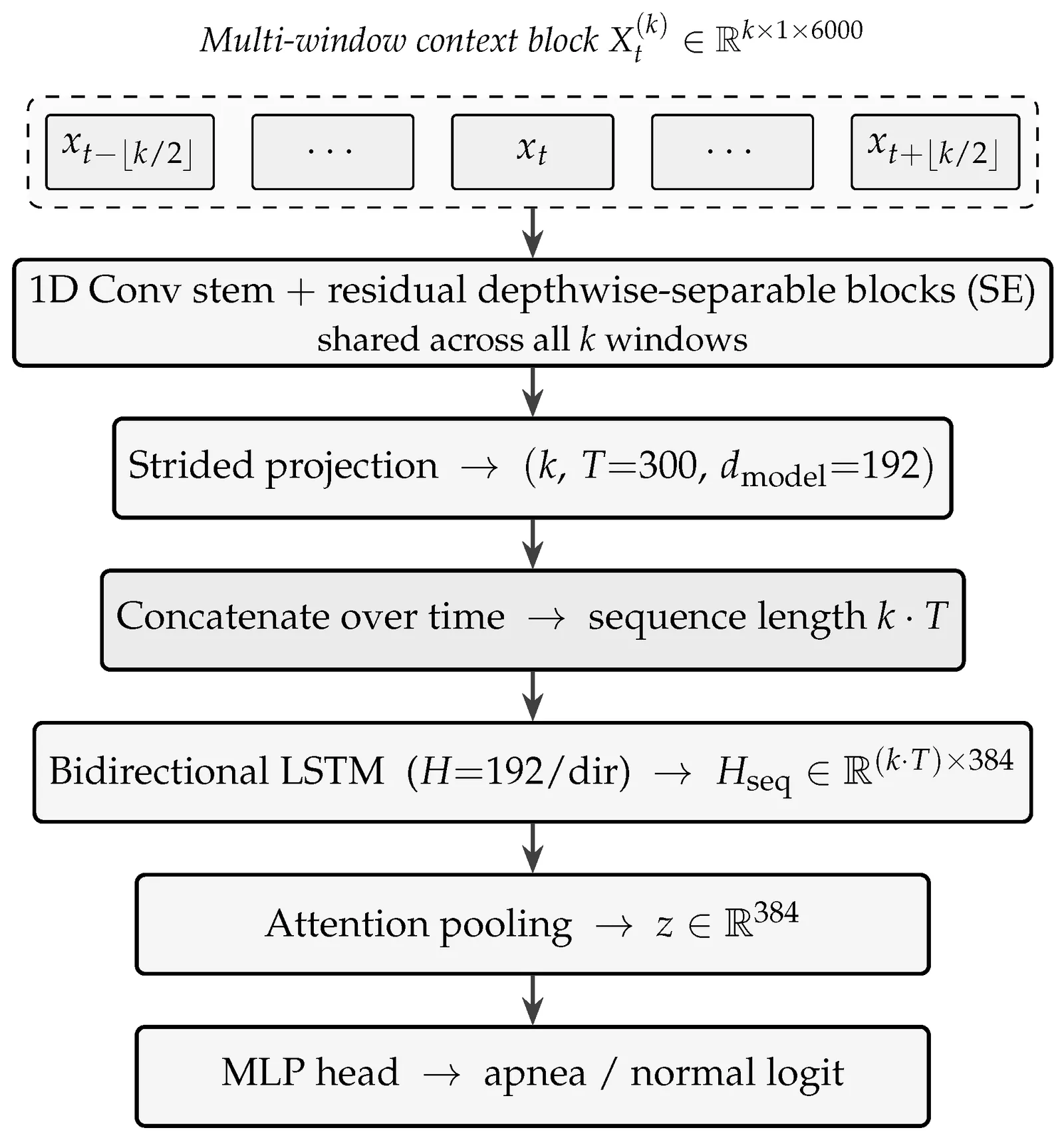
Overview of the proposed multi-window architecture. The *k* adjacent 1-min segments comprising the context block $X_{t}^{\left(k\right)}$ are first processed using a shared 1D convolutional stem. The resulting feature representations are then concatenated along the temporal dimension to form a single sequence of length k·T, which is subsequently modeled using a bidirectional LSTM. Finally, attention pooling and an MLP classification head are applied to generate the prediction. SE denotes squeeze-and-excitation.

The overall network architecture is illustrated in Figure 1. The backbone was intentionally designed as a conventional, general-purpose CNN–BiLSTM architecture rather than a highly optimized or state-of-the-art model. Because the primary objective of this study was to isolate the effect of temporal context length (*k*), the network architecture was held constant across all experimental configurations and was based largely on the single-window framework described in our previous study [10]. Only the input representation and sequence-processing components were modified to accommodate multi-window context blocks. This design helps isolate performance differences associated with the incorporation of adjacent temporal context from those attributable to architecture-specific tuning.

The model receives a context tensor of shape (B,k,1,6000), which is reshaped to (B·k,1,6000) so that all constituent windows are processed by the same convolutional encoder. The resulting signals are passed through a two-stage 1D convolutional stem to extract low-level local P–QRS–T morphological features while performing initial temporal compression:

(2) $$\begin{array}{cc} f_{1} & =\mathrm{SiLU}(\mathrm{BN}(\mathrm{Conv}1D(1\to 64,\mathrm{kernel}=15,\mathrm{stride}=2))) \end{array}$$

(3) $$\begin{array}{cc} f_{2} & =\mathrm{SiLU}(\mathrm{BN}(\mathrm{Conv}1D(64\to 128,\mathrm{kernel}=11,\mathrm{stride}=2))) \end{array}$$

The convolutional stem is followed by two residual depthwise-separable convolutional blocks (with kernel sizes of 9 and 7) augmented with squeeze-and-excitation gating to adaptively recalibrate channel responses. Temporal resolution is then reduced through a strided convolution (kernel=5, stride=5), producing a compact feature sequence of length T=300 with $d_{\mathrm{model}}=192$ channels. The feature representations extracted from each context window were concatenated along the temporal dimension to form a single sequence of length k·T. This sequence was subsequently processed by a single-layer bidirectional LSTM (H=192 per direction), producing contextualized outputs, $H_{\mathrm{seq}}\in R^{B\times (k\cdot T)\times 384}$. Rather than using average pooling, a trainable attention mechanism was employed to compute softmax-normalized pooling weights, $\alpha_{t}$, enabling the aggregation of the most informative features into a dense representation, $z\in R^{384}$:

(4) $$\alpha_{t}=\frac{\exp(W_{\mathrm{att}}H_{t})}{\sum_{j=1}^{k\cdot T}\exp\left(W_{\mathrm{att}}H_{j}\right)},\;z=\sum_{t=1}^{k\cdot T}\alpha_{t}H_{t}$$

The pooled representation was mapped to a segment-level output logit through a two-layer MLP head incorporating intermediate dropout and SiLU activation functions. During training, an exponential moving average (EMA) of the network parameters (γ=0.999) was maintained and employed for validation and test inference to mitigate sensitivity to short-term stochastic parameter fluctuations, yielding more stable performance estimates. The classification threshold was recalibrated at each training epoch by evaluating 81 uniformly spaced candidate thresholds from 0.10 to 0.90 in increments of 0.01 and selecting the value that maximized the validation F1-score. To reduce epoch-to-epoch variability, the selected threshold was smoothed using a five-epoch running average before being applied, without modification, to the test set. For the primary analysis, the checkpoint yielding the highest validation F1-score was selected for each experimental run; neither checkpoint selection nor threshold calibration used test-set performance. Performance at a fixed classification threshold of 0.5 was additionally evaluated as a robustness analysis. Consistent with our previous protocol [10], the apneic class was treated as the positive class when computing F1-score, precision, and recall, and area under the receiver operating characteristic curve (AUROC) and area under the precision–recall curve (AUPRC) were calculated accordingly. Complete optimization, training, and model-selection settings are provided in Supplementary Table S6. Representative training and validation loss trajectories, together with the distribution of validation-F1-selected checkpoint epochs, are provided in Supplementary Figure S2.

### 3.7. Statistical Analysis

The primary outcome was the test-set F1-score, with AUPRC, AUROC, and accuracy treated as secondary performance measures. For each of the 35 combinations of apnea-label retention ratio (*r*) and context length (*k*), performance metrics were summarized across three training repetitions and five participant-grouped folds (n=15 runs per condition).

The effects of context length, apnea-label retention, and their interaction were evaluated using factorial models that included fold and training repetition as blocking factors. Because repeated training runs within the same fold shared the same test participants, primary inferential *p*-values were obtained using 10,000 restricted Freedman–Lane residual permutations, with the same condition-wise residual permutation applied jointly across all three repetitions within each fold. Permutations were restricted within fold-by-*r* strata for the effect of *k*, within fold-by-*k* strata for the effect of *r*, and across the complete r×k condition matrix within each fold for the interaction. Reference factorial ANOVA models were additionally used to report *F* statistics and partial $\eta^{2}$ effect sizes.

Planned comparisons evaluated each multi-window configuration (k=3,5,7,9) against the corresponding single-window condition (k=1) at each value of *r*, yielding 28 paired contrasts. For these comparisons, paired differences in F1-score (ΔF1) were reported with 95% confidence intervals obtained from 10,000 two-way crossed bootstrap resamples, in which training repetitions and participant-grouped folds were independently resampled with replacement. As a sensitivity analysis, the three repetitions were first averaged within each fold and exact one-sided sign-flip tests were then performed across the five fold-level differences; the resulting 28 *p*-values were adjusted using the Holm procedure. Statistical significance was assessed at α=0.05.

## 4. Results

### 4.1. Performance Dynamics Across Apnea-Label Retention Levels

**Figure 2:**
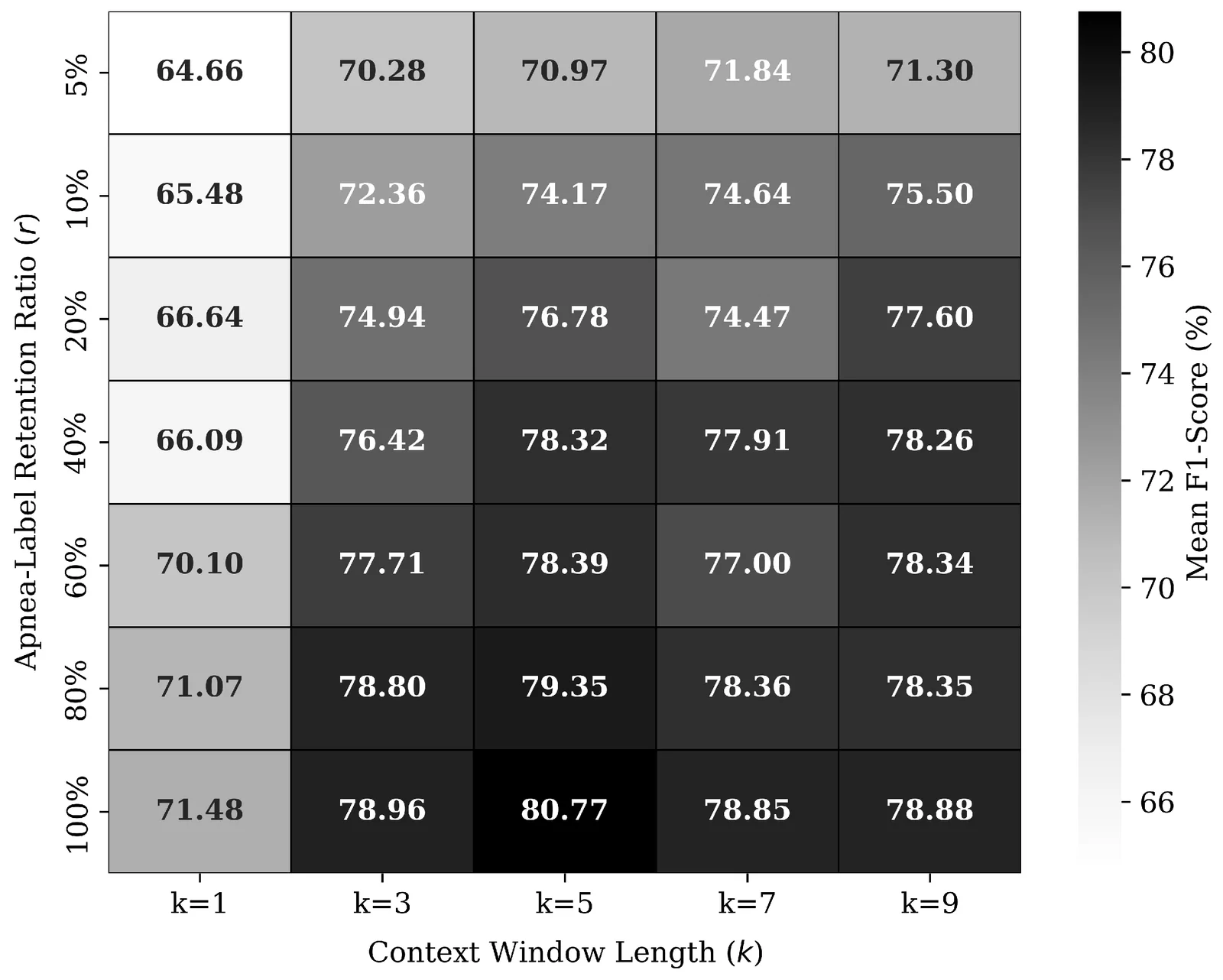
Grayscale heatmap of the mean test-set F1-scores across the 35 experimental configurations. Values represent the mean test-set F1-scores (%) across three training repetitions and five participant-grouped folds (n=15 runs per condition) for different apnea-label retention ratios (*r*) and context window lengths (*k*). Darker shading indicates higher performance.

**Figure 3:**
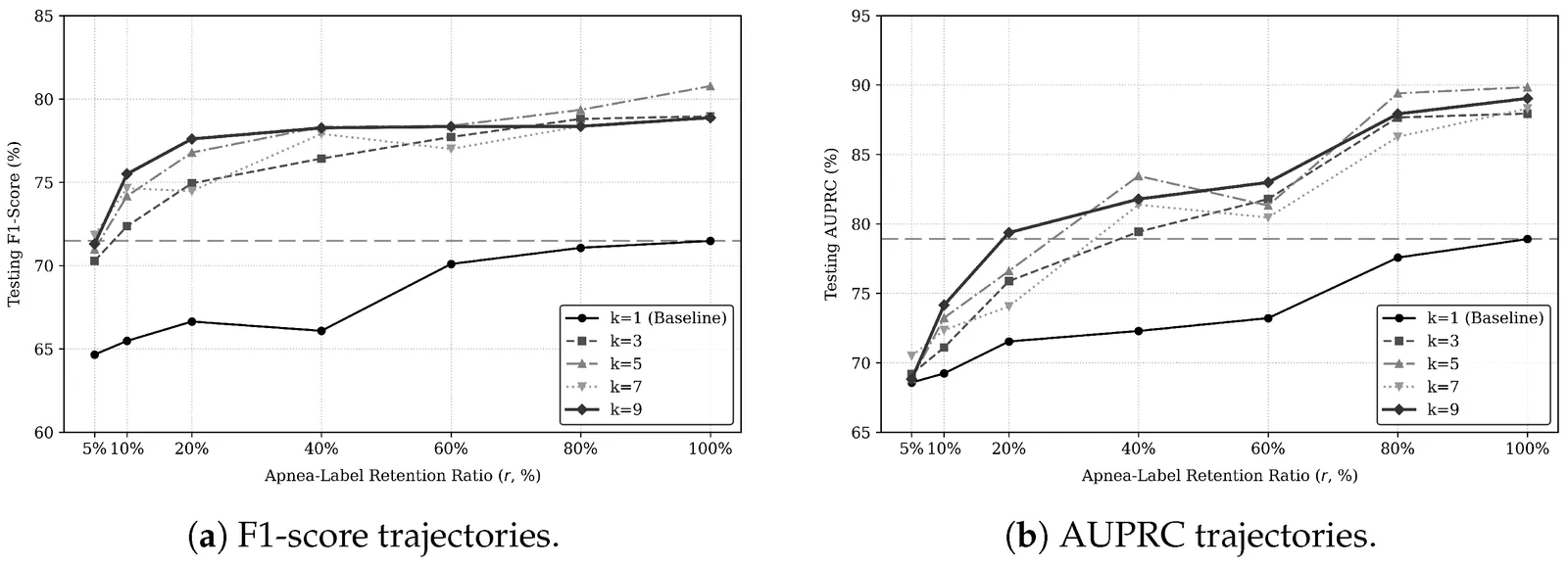
Test-set performance trajectories across apnea-label retention levels for the full range of context window lengths (k∈{1,3,5,7,9}). Panel (**a**) presents the test-set F1-score, whereas Panel (**b**) presents the corresponding test-set AUPRC. Markers represent mean performance across the 15 runs in each experimental condition. In both panels, the horizontal reference line indicates the corresponding full-retention single-window baseline (r=100%, k=1).

The results across the 35 experimental configurations demonstrated a consistent descriptive advantage of multi-window context across the full range of apnea-label retention levels. As illustrated in Figure 2 and Figure 3a, all four multi-window configurations (k>1) yielded higher mean F1-scores than the corresponding single-window baseline (k=1) at every value of *r*. However, the improvement was not monotonic with respect to context length, and the context length yielding the highest mean F1-score varied across retention levels.

Table 1 summarizes the single-window baseline and the best-performing multi-window configuration at each apnea-label retention level. Under the most restrictive apnea-label retention condition (r=5%), the single-window baseline achieved a mean F1-score of 64.66%±5.55%, whereas the highest mean F1-score was obtained at k=7 (71.84%±5.97%), corresponding to a gain of +7.18 percentage points. At r=10% and 20%, the highest mean F1-scores were obtained at k=9, whereas k=5 yielded the highest mean F1-score at r=40%, 60%, 80%, and 100%. Across the seven retention levels, the best multi-window configuration improved mean F1-score by 7.18–12.23 percentage points compared with the corresponding k=1 condition, equivalent to relative gains of approximately 11.1–18.5%. Under full apnea-label retention (r=100%), the highest mean F1-score was 80.77%±5.10% at k=5, compared with 71.48%±8.85% for k=1. Relative to the full-retention single-window reference (r=100%, k=1; 71.48%), the best multi-window configurations at r=5% and 10% achieved mean F1-scores of 71.84% and 75.50%, respectively; these descriptive crossovers are visualized directly in Figure 3a by the horizontal reference line.

As illustrated in Figure 3b, AUPRC showed a broadly similar but less pronounced pattern, particularly at r=5%, where increasing temporal context produced only modest changes in mean AUPRC. Mean AUROC and accuracy values likewise exceeded their corresponding single-window values in all 28 multi-window comparisons. Complete descriptive results for all 35 experimental conditions are provided in Supplementary Table S7. Representative aggregated confusion matrices comparing the single-window baseline with the best-performing multi-window configuration at selected apnea-label retention levels are provided in Supplementary Figure S1.

### 4.2. Statistical Effects of Context Length and Apnea-Label Retention

The factorial analysis provided no evidence of a systematic interaction between context length and apnea-label retention ratio (k×r: F(24,484)=0.54, permutation p=0.9997, partial $\eta^{2}=0.026$), indicating no detectable systematic variation in the effect of temporal context across the evaluated retention levels. Given the absence of evidence for an interaction effect, the main effects of *k* and *r* were summarized using the additive model. Context length showed a clear overall effect on test-set F1-score (F(4,508)=55.13, permutation p=0.0001, partial $\eta^{2}=0.303$), as did apnea-label retention ratio (F(6,508)=21.56, permutation p=0.0001, partial $\eta^{2}=0.203$). The omnibus statistical results are summarized in Table 2.

Planned paired comparisons further quantified the effect of each multi-window configuration relative to the corresponding single-window baseline. All 28 comparisons yielded positive mean F1-score differences, with improvements ranging from +5.62 to +12.23 percentage points. The two-way crossed bootstrap 95% confidence intervals excluded zero in 27 of the 28 comparisons. The sole exception was the r=5%, k=9 comparison, which showed a mean improvement of +6.64 percentage points but a wider 95% confidence interval spanning −2.66 to +14.45 percentage points.

After averaging the three training repetitions within each participant-grouped fold, 20 of the 28 comparisons showed positive F1-score differences in all five folds. In the exact one-sided sign-flip sensitivity analysis, the smallest attainable raw *p*-value with five fold-level observations was 0.03125 because only $2^{5}=32$ possible sign configurations exist. Consequently, none of the 28 comparisons remained significant after Holm adjustment (minimum adjusted p=0.875). Complete effect estimates, crossed-bootstrap confidence intervals, fold-wise direction consistency, and exact sign-flip sensitivity results are provided in Supplementary Table S8.

### 4.3. Fixed-Threshold Robustness Analysis

To assess whether the observed multi-window advantage depended on validation-specific threshold optimization, the selected EMA checkpoint from each experimental run was additionally evaluated on the test set using a fixed classification threshold of 0.5. Mean F1-score differences remained positive in all 28 multi-window comparisons relative to the corresponding k=1 baseline under the fixed threshold, with gains ranging from +1.09 to +12.29 percentage points. For example, at r=10%, fixed-threshold F1 increased from 63.99%±8.82% at k=1 to 69.39%±5.42% at k=9; at r=40%, from 64.67%±11.72% at k=1 to 76.96%±5.78% at k=5; and at r=100%, from 71.00%±9.47% at k=1 to 80.11%±4.12% at k=5. These findings indicate that the qualitative advantage of multi-window context was preserved when test predictions were evaluated using a common fixed threshold of 0.5. Complete fixed-threshold performance results are provided in Supplementary Table S10.

### 4.4. Control Analysis of Adjacent Temporal Information

To examine whether the observed performance improvement was attributable specifically to information contained in adjacent ECG segments, we conducted targeted control experiments using representative configurations selected from the full experimental matrix. The control analysis focused on the k=9 setting at r=5%, 10%, and 100%, spanning low and full apnea-label retention conditions. Two control configurations were evaluated: a repeated-center control in which the central 60-s target epoch was replicated nine times, and a random-shuffle control in which the eight neighboring positions were filled with epochs randomly sampled from the same ECG recording but located at least 5 min away from the central target epoch, thereby preserving the recording source and overall input length while disrupting local temporal adjacency. All control conditions were evaluated using the same three training repetitions and five participant-grouped folds as the corresponding adjacent-context experiments (n=15 runs per condition).

As summarized in Table 3, neither control configuration reproduced the performance obtained using truly adjacent temporal context. The adjacent k=9 configuration exceeded the repeated-center control by 12.31, 9.64, and 9.44 percentage points at r=5%, 10%, and 100%, respectively, and yielded a higher F1-score in 42 of the 45 paired runs. The contrast was even larger for the random-shuffle control: adjacent context improved mean F1-score by 21.51, 18.90, and 21.55 percentage points at r=5%, 10%, and 100%, respectively, and yielded a higher F1-score in all 45 paired runs. After averaging across retention levels and training repetitions, the adjacent-minus-control difference was positive in all five participant-grouped folds for both the repeated-center and random-shuffle comparisons. These results indicate that the benefit of the adjacent k=9 configuration cannot be explained by sequence-length expansion, repeated presentation of the central target epoch, or the addition of temporally distant ECG segments from the same recording alone, and instead support a specific contribution from locally adjacent temporal information.

## 5. Discussion

The findings of this study provide insight into data-efficient sequence modeling under conditions of limited supervision. A key observation from the performance matrix (Table 1 and Supplementary Table S7) is that all multi-window configurations achieved higher mean F1-scores than the corresponding single-window baseline across the full range of apnea-label retention levels. The factorial analysis further demonstrated a clear overall effect of context length, whereas no evidence of a systematic interaction between context length and apnea-label retention was observed. These findings indicate that the benefit of temporal context was not confined to conditions of severe apnea-label scarcity but was broadly maintained across the evaluated supervision range.

### 5.1. Contribution of Adjacent Temporal Context

With the multi-window configuration, the network is encouraged to capture continuous temporal state transitions rather than relying solely on isolated epoch-level variations. Across the 35 experimental configurations, all 28 multi-window comparisons showed positive mean F1-score differences relative to the corresponding k=1 baseline, with the best-performing multi-window configuration at each retention level providing gains of 7.18–12.23 percentage points. However, the improvement was not monotonic with respect to context length, and the context length associated with the highest mean F1-score varied across apnea-label retention levels. This pattern suggests that the benefit of multi-window modeling does not arise simply from increasing the temporal extent of the input, but rather from incorporating useful information surrounding the target epoch. Because these observations were obtained using a single benchmark dataset and one CNN–BiLSTM backbone, they should not be interpreted as evidence of a general performance plateau or saturation point for temporal context.

The targeted control experiments provided additional support for this interpretation. When the central target epoch was replicated nine times, the repeated-center configuration failed to reproduce the performance gain obtained with the true adjacent k=9 sequence. Similarly, replacing the eight neighboring epochs with windows randomly sampled from the same ECG recording but located at least 5 min away from the central target epoch did not reproduce the performance of the adjacent-context configuration. Because the random-shuffle control preserved the participant and recording source while disrupting local temporal adjacency, the contrast between these conditions suggests that the observed gain cannot be explained solely by increased sequence length, repeated presentation of the target epoch, or the inclusion of temporally distant ECG segments from the same recording. Rather, the results support a contribution from information specifically contained in the temporally adjacent ECG segments.

In clinical applications such as sleep and cardiorespiratory monitoring, subject-independent generalization is often challenged by substantial inter-patient biological variability. Localized temporal context may provide complementary information about evolving cardiorespiratory patterns surrounding an individual 60-s target epoch, thereby reducing reliance on features contained within a single isolated segment. This interpretation is consistent with evidence from the broader time-series literature indicating that adjacent-window aggregation and temporal context can reduce sensitivity to stochastic local fluctuations and improve anomaly discrimination [29,30].

To contextualize the absolute performance of the proposed framework, Table 4 summarizes representative ECG-based apnea detection studies that incorporate recurrent, attention-based, extended-neighborhood, or other temporal modeling strategies. Several previous approaches have reported higher absolute per-segment performance on the Apnea-ECG database; however, direct numerical ranking should be interpreted cautiously because these studies differ substantially in recording or participant partitioning, input representation, temporal context, preprocessing, and model-selection procedures. In particular, the present study used conservative participant-grouped five-fold cross-validation and was designed to isolate the effect of adjacent temporal context rather than to optimize a state-of-the-art architecture. The comparison is therefore provided to contextualize the present results within the broader ECG-based sequence-modeling literature rather than to support claims of direct performance superiority.

### 5.2. Data Efficiency Under Limited Apnea-Label Retention

A key practical observation emerges from the performance trajectories shown in Figure 3. The benefit of adjacent temporal context was maintained even when only a small proportion of real apnea target labels was retained for training. At r=5%, the best-performing multi-window configuration (k=7) achieved a mean F1-score of 71.84%, compared with 64.66% for the corresponding k=1 condition. At r=10%, k=9 achieved 75.50%, exceeding both the corresponding k=1 condition (65.48%) and the fully retained single-window condition at r=100% (71.48%). A similar descriptive crossover was observed at r=5%, where the best multi-window mean F1-score was slightly higher than that of the r=100%, k=1 reference.

In clinical settings where expert-provided apnea annotations are limited by the substantial effort required for manual labeling, these findings suggest that leveraging neighboring ECG segments can improve the utility of a reduced set of real apnea target labels. By incorporating information from readily available neighboring segments as contextual input, the framework extracts additional value from existing records without requiring the neighboring epochs to provide additional supervised target labels. Importantly, however, *r* in the present study denotes the fraction of real apnea target labels retained rather than the fraction of all training annotations retained; all normal target labels remained available, and offline augmentation restored the effective positive-class sample count. The observed crossovers should therefore be interpreted as evidence of robustness to reduced apnea-label availability rather than as a direct quantitative estimate of the overall reduction in clinical annotation workload. Because these findings were obtained using the Apnea-ECG dataset, CNN–BiLSTM architecture, participant-grouped evaluation protocol, and augmentation framework employed in this study, they should be regarded as suggestive rather than as a general estimate of annotation savings. Although deployment-oriented models would generally be developed using as much reliable labeled data as feasible, the lower-retention conditions remain practically relevant to early-stage or resource-constrained model development in which dense apnea annotations are not available. This observation is consistent with findings from recent studies on data-efficient time-series representation learning [20], suggesting that leveraging temporal context may reduce dependence on dense positive-label supervision without relying on extensive cross-domain pre-training [21].

### 5.3. Statistical Evidence and Consistency of the Context Effect

From a statistical perspective, the omnibus analysis provided strong evidence that context length contributed to test-set F1-score across the complete experimental matrix. The main effect of *k* was supported by the restricted permutation analysis (F(4,508)=55.13, permutation p=0.0001, partial $\eta^{2}=0.303$), whereas the k×r interaction was small and unsupported (F(24,484)=0.54, permutation p=0.9997, partial $\eta^{2}=0.026$). Together with the uniformly positive mean differences observed across all 28 planned multi-window comparisons, these results indicate that the advantage of temporal context was broadly consistent across apnea-label retention levels rather than being driven by a specific supervision regime.

The planned comparisons provided further information about the magnitude and stability of these effects. Two-way crossed-bootstrap 95% confidence intervals excluded zero in 27 of the 28 comparisons, with the only exception occurring for r=5% and k=9, where between-fold and between-repetition variability produced a wider interval. After averaging repetitions within each participant-grouped fold, 20 of the 28 comparisons remained positive in all five folds. The exact sign-flip sensitivity analysis was necessarily limited by the five available fold-level observations, for which the smallest attainable one-sided raw *p*-value was 0.03125; after adjustment for 28 comparisons, none remained significant under the Holm procedure. Accordingly, the fold-level sensitivity analysis should be interpreted as a conservative assessment of directional consistency rather than as the primary inferential basis for each individual *k*-versus-1 contrast. The overall evidence is therefore best characterized by the permutation-supported main effect of context length, the consistently positive effect estimates, and the predominance of bootstrap intervals excluding zero, while avoiding claims that every individual multi-window configuration was independently significant.

### 5.4. Potential Clinical Implications

Beyond the quantitative performance gains, the data-efficient behavior observed here may have practical implications for ECG-based screening workflows. Rather than the absolute level of performance achieved, the key observation is that using multi-window temporal context consistently improved performance relative to the single-window baseline across the full range of apnea-label retention levels. These findings suggest that, in settings where dense expert-provided apnea annotations are limited, leveraging adjacent temporal context may help reduce dependence on dense apnea-target supervision during model development. By incorporating neighboring ECG segments that are already available within the continuous recording, the proposed approach increases the amount of physiological context available to the classifier without requiring additional labels for those neighboring epochs. Because the retention ratio *r* applies only to real apnea target labels while all normal target labels remain available, the present experimental design does not support a direct conversion of *r* into expert annotation hours; prospective workflow studies measuring actual labeling time would be required to quantify such savings.

In a potential screening application, such a context-enhanced model could process overnight single-lead ECG recordings, flag segments with elevated predicted apnea risk for expert review, and support first-pass triage of individuals requiring subsequent confirmatory assessment, rather than serving as a stand-alone diagnostic system. The present findings are therefore more relevant to improving the efficiency and robustness of ECG-based screening model development than to establishing direct diagnostic equivalence with clinical sleep studies. Importantly, the control experiments further suggest that this benefit is associated with temporally adjacent ECG information rather than simply with longer input sequences or additional same-recording segments, supporting the practical value of preserving local temporal structure when designing ECG-based screening systems. Before clinical deployment, the framework would require recording-level aggregation and calibration to derive subject-level AHI or clinically defined severity categories, external validation across independent and device-diverse cohorts, and prospective evaluation against established sleep-study reference standards using clinically relevant subject-level screening endpoints.

### 5.5. Limitations and Future Work

Several limitations should be considered when interpreting these results. All experiments were conducted using a single public benchmark dataset, the PhysioNet Apnea-ECG database, which includes a relatively limited number of participant groups and was collected under controlled recording conditions. Consequently, the generalizability of the observed trends to larger and more heterogeneous cohorts, as well as to signals acquired using consumer-grade wearable devices, remains to be established. Wearable ECG may exhibit lower or more variable signal quality because of motion artifacts, electrode-contact variability, and device-specific acquisition characteristics, which could affect the magnitude of the performance gains observed under the controlled benchmark conditions. External validation using independent datasets therefore represents an important direction for future work.

The task was formulated as a binary segment-level classification problem distinguishing apneic from normal epochs and did not include estimation of subject-level severity metrics, such as the apnea–hypopnea index, which play a central role in clinical diagnosis. Extending the proposed multi-window framework to support severity estimation therefore represents a logical direction for future investigation. Such an extension could aggregate segment-level predictions across an overnight recording to derive subject-level event rates and severity categories, but would require reliable event counting, recording-level calibration, and validation against clinically defined severity thresholds. If the reduction in segment-level classification errors observed with temporal context is maintained across overnight recordings, it could potentially improve the stability of recording-level event-rate or severity estimates; however, this was not evaluated directly in the present study. Prospective evaluation using subject-level diagnostic endpoints will also be required before the observed segment-level improvements can be translated into clinical screening performance.

The apnea-label retention protocol also warrants careful interpretation. The retention ratio *r* refers specifically to the proportion of real apnea target labels retained for training rather than to the proportion of all training annotations, because all normal target labels remained available. Moreover, for r<100%, offline augmentation restored the effective positive-class sample count to that of the full-retention condition. Accordingly, performance under low-retention conditions reflects learning with reduced real apnea supervision in combination with the fixed augmentation strategy rather than the effect of label removal in isolation. Consequently, the effective apnea-class training pool in the most restrictive conditions was dominated by offline-augmented samples, particularly at r=5% and 10%. Although these transformations increase variability around the retained real apnea segments, they do not introduce independent participants and may not reproduce the full patient-level, device-specific, or acquisition-related heterogeneity encountered in genuinely scarce clinical datasets. The performance observed under these simulated low-retention conditions should therefore not be assumed to transfer directly to clinical datasets with scarce labels that are not supplemented by a comparable augmentation strategy, and external validation in such settings remains necessary. Because the augmentation protocol was held constant across context lengths within each retention condition, this design does not confound the paired evaluation of *k*; however, the values of *r* should not be interpreted as direct estimates of proportional reductions in total clinical annotation workload.

The present study additionally evaluated temporal context using a single CNN–BiLSTM backbone. Although fixing the architecture across experimental conditions was necessary to isolate the effect of context length, the extent to which the observed benefit generalizes to other sequence-modeling architectures remains to be determined. Evaluation with alternative convolutional, attention-based, and state-space models would therefore help establish whether adjacent temporal context provides an architecture-independent advantage.

The repeated-center and random-shuffle control experiments strengthen the interpretation that locally adjacent ECG segments provide information beyond that obtained from longer input sequences, repeated target presentation, or temporally distant same-recording ECG segments. However, these controls do not identify the specific physiological features responsible for this benefit. Further analyses of heart-rate dynamics, respiration-modulated ECG characteristics, temporal event transitions, and model attention patterns may help clarify which components of the neighboring segments contribute most strongly to central-epoch classification. In addition, although participant-level grouping prevented cross-partition leakage, the relatively small cohort was represented by only five outer folds, which limited the resolution of fold-level exact sensitivity testing. Larger independent cohorts would permit more precise participant-level estimation of individual context effects and stronger confirmatory inference for pairwise comparisons.

Increasing the context length expands the effective input sequence to k·T, thus increasing the computational and memory demands of the recurrent component, even though the overall parameter count remains unchanged. This trade-off is particularly relevant for deployment on resource-constrained wearable platforms and warrants further evaluation. Future work should therefore examine the trade-off between context duration, predictive performance, and inference cost, including adaptive strategies that select only the temporal context required for a given target epoch.

## 6. Conclusions

Here, we systematically investigated the interaction between context window length (*k*) and apnea-label retention (*r*) within a multi-window sequence modeling framework. Evaluation across 35 experimental configurations using participant-grouped five-fold cross-validation demonstrated a consistent advantage of multi-window temporal context over the corresponding single-window baseline across the full range of apnea-label retention levels. Context length showed a clear overall effect on test-set F1-score, whereas no evidence of a systematic interaction between context length and apnea-label retention was observed, indicating that the benefit of temporal context was broadly maintained across different levels of supervision.

The best-performing multi-window configuration improved mean F1-score by 7.18–12.23 percentage points relative to the corresponding k=1 condition across the seven retention levels. Targeted control experiments further supported the contribution of local temporal information, as neither repeated presentation of the central epoch nor temporally distant same-recording ECG segments reproduced the performance obtained using truly adjacent context. In conclusion, the proposed framework shows that leveraging readily available contiguous temporal sequences can improve ECG-based apnea classification while reducing dependence on dense real apnea-label supervision. These findings support adjacent temporal context as a simple and potentially useful design strategy for ECG-based sleep apnea modeling, while further validation in larger, independent cohorts and alternative model architectures remains necessary.

## Figures and Tables

**Table 1:** Condensed summary of test-set F1-scores across apnea-label retention levels. Values are reported as means ± SD across three training repetitions and five participant-grouped folds (n=15 runs per condition). For each value of *r*, the multi-window configuration with the highest mean F1-score is shown together with the corresponding single-window baseline.

*r* (%)	F1 (%) at k=1	Best Multi-Window *k*	F1 (%) at Best *k*	ΔF1 (pp)
5	64.66±5.55	7	71.84±5.97	+7.18
10	65.48±8.09	9	75.50±3.56	+10.02
20	66.64±6.50	9	77.60±5.18	+10.96
40	66.09±10.76	5	78.32±4.27	+12.23
60	70.10±8.18	5	78.39±4.76	+8.29
80	71.07±7.95	5	79.35±4.48	+8.28
100	71.48±8.85	5	80.77±5.10	+9.29

**Table 2:** Omnibus statistical analysis of test-set F1-score. Permutation *p*-values were obtained using 10,000 restricted Freedman–Lane residual permutations. *F* statistics and partial $\eta^{2}$ effect sizes for the main effects of *k* and *r* were obtained from the additive model, whereas the interaction was evaluated using the full factorial model.

Effect	*F* Statistic	Permutation *p*	Partial $\eta^{2}$
Context length (*k*)	F(4,508)=55.13	0.0001	0.303
Apnea-label retention (*r*)	F(6,508)=21.56	0.0001	0.203
k×r interaction	F(24,484)=0.54	0.9997	0.026

**Table 3:** Test-set F1-score for the repeated-center and random-shuffle control experiments. Values are reported as means ± SD across three training repetitions and five participant-grouped folds (n=15 runs per condition). ΔF1 denotes the paired mean difference between the adjacent k=9 configuration and the corresponding control.

*r* (%)	k=1	Repeated Center (k=9)	Random Shuffle (k=9)	Adjacent (k=9)	ΔF1 vs. Repeated	ΔF1 vs. Random
5	64.66±5.55	58.99±6.76	49.78±12.70	71.30±7.03	+12.31	+21.51
10	65.48±8.09	65.86±6.09	56.59±7.80	75.50±3.56	+9.64	+18.90
100	71.48±8.85	69.44±9.72	57.33±4.96	78.88±3.96	+9.44	+21.55

**Table 4:** Representative comparison with previous ECG-based sleep apnea detection studies incorporating temporal or contextual information. Reported values correspond to per-segment classification performance on the PhysioNet Apnea-ECG database.

Study	Model	Evaluation/Temporal Setting	Accuracy (%)	F1 (%)
Almutairi et al. (2021) [7]	CNN–LSTM	Released/withheld 35/35 recording split; recurrent modeling of ECG-derived features	90.92	92.76
Hu et al. (2022) [14]	Hybrid Transformer	35/35 recording split; 6-min temporal input with self-attention	90.52	87.47
Li et al. (2024) [34]	EDSFnet	35/35 recording split; extended ECG segments used to incorporate neighborhood information	92.60	–
Zhao et al. (2024) [35]	DM-IACNN	Apnea-ECG; multi-scale inputs constructed from adjacent ECG segments	91.02	87.60
Pham and Mouček (2025) [15]	CNN–Transformer–LSTM	Hold-out validation; temporal modeling of R–R intervals and R-peak-derived inputs	91.60	89.00
Zan (2025) [36]	CNN–S4	Apnea-ECG; structured state-space modeling of long-range temporal dependencies	93.30	91.20
Present study	CNN–BiLSTM	Participant-grouped five-fold cross-validation with three repetitions; adjacent 5-min context at r=100%	85.09	80.77

Reported values are reproduced from the respective studies for contextual comparison only. The evaluation protocols are not directly equivalent, particularly with respect to recording versus participant grouping, temporal input construction, preprocessing, feature representation, and model-selection procedures. Values for the present study correspond to the highest mean test-set F1-score under full apnea-label retention (r=100%, k=5). A dash indicates that the corresponding metric was not reported in the principal per-segment result used for this comparison.

## Data Availability

The data presented in this study are available in the PhysioNet Apnea-ECG Database (v1.0.0) at https://doi.org/10.13026/C23W2R. These data were derived from the following resource available in the public domain: PhysioNet Apnea-ECG Database, https://physionet.org/content/apnea-ecg/1.0.0/ (accessed on 24 August 2026).

## Supplementary Materials

The following supporting information can be downloaded at https://www.mdpi.com/article/10.3390/diagnostics16182939/s1, Table S1: Signal quality-control and shared-anchor construction statistics; Table S2: Participant-level grouping and outer-fold assignment; Table S3: Participant and class balance across the five outer cross-validation folds; Table S4: Fold-specific real apnea-label retention and offline augmentation counts used in the training partitions; Table S5: Offline and online augmentation settings applied to each constituent 60-s ECG window; Table S6: Complete training, optimization, and model-selection settings; Figure S1: Representative aggregated confusion matrices for selected single-window and multi-window conditions; Figure S2: Representative training dynamics for selected multi-window configurations; Figure S3: Representative pooled mean-beat morphology under low apnea-label retention; Table S7: Complete test-set performance across the 35 combinations of apnea-label retention ratio and context length; Table S8: Planned F1-score comparisons between multi-window configurations and the corresponding single-window baseline; Table S9: Apnea-related exposure through unlabeled neighboring context under low apnea-label retention; Table S10: Test-set performance in the fixed-threshold robustness analysis.

## Abbreviations

The following abbreviations are used in this manuscript:

AHI	Apnea–Hypopnea Index
ANOVA	Analysis of Variance
AUPRC	Area Under the Precision–Recall Curve
AUROC	Area Under the Receiver Operating Characteristic Curve
BN	Batch Normalization
BiLSTM	Bidirectional Long Short-Term Memory
CI	Confidence Interval
CNN	Convolutional Neural Network
ECG	Electrocardiography
EMA	Exponential Moving Average
HRV	Heart Rate Variability
MLP	Multilayer Perceptron
OSA	Obstructive Sleep Apnea
PSG	Polysomnography
SD	Standard Deviation
SiLU	Sigmoid Linear Unit
