# Supplementary material for: Multi-Window Temporal Context for ECG-Based Sleep Apnea Detection Under Limited Apnea-Label Availability
Source: Diagnostics (Basel). 2026 Sep 11;16(18):2939. doi: 10.3390/diagnostics16182939 (PMC13605058; doi:10.3390/diagnostics16182939)
Supplement: Supplementary file 1 [file diagnostics-16-02939-s001.zip › diagnostics-4417173-supplementary.pdf]

Supplementary Materials

Multi-Window Temporal Context for ECG-Based Sleep Apnea Detection under Limited Apnea-Label Availability

Semin Ryu[1,2,*], Jeonghwan Koh[1,2], and In cheol Jeong[1,2,3,*]

[1] Department of Artificial Intelligence Convergence, Hallym University, Chuncheon 24252, Republic of Korea; jeonghwan.koh@hallym.ac.kr
[2] Cerebrovascular Disease Research Center, Hallym University, Chuncheon 24252, Republic of Korea
[3] Department of Population Health Science and Policy, Icahn School of Medicine, Mount Sinai, New York, NY 10029, USA
[*] Correspondence: sr@hallym.ac.kr (S.R.); incheol.jeong@hallym.ac.kr (I.c.J.)

Article Title: Multi-Window Temporal Context for ECG-Based Sleep Apnea Detection under Limited Apnea-Label Availability

Journal: Diagnostics

Supplementary Materials: This supplementary file contains additional information supporting the main manuscript, including supplementary methods, results, figures, and tables.

Supplementary Methods

Signal Preprocessing and Shared Anchor Construction

To ensure identical target-anchor composition across all temporal context lengths, eligible target epochs were defined using the most restrictive context configuration ($k = 9$). Signal quality-control and shared-anchor construction statistics are summarized in Table S1. Briefly, individual 60-s windows that were truncated, non-finite, or near-flat were considered invalid, and candidate anchors were retained only when the complete nine-epoch context consisted of valid and temporally continuous windows. Anchor eligibility was determined solely by recording position, signal validity, and temporal continuity rather than by the apnea/normal label. Because a single invalid 60-s window can affect multiple neighboring candidate anchors, the numbers of invalid individual windows and rejected candidate anchors represent different counting units. However, epochs excluded from the final shared target-anchor set were not separately characterized, and systematic differences between the excluded and retained epochs therefore cannot be ruled out.

Table S1: Signal quality-control and shared-anchor construction statistics.

**Quantity**	**Count**
Included ECG recordings	69
Excluded ECG recordings	1 (c05)
Original 60-s annotations	33,847
Valid individual windows	33,773
Invalid individual windows	74
Truncated tail windows	41
Non-finite windows	0
Near-flat windows	33
Recording-edge annotations excluded for $k = 9$ context	552
Candidate target anchors	33,295
Candidate anchors rejected because of one or more invalid constituent windows	164
Candidate anchors rejected because of temporal discontinuity	0
Final shared target anchors	33,131
Normal anchors	20,208
Apneic anchors	12,923
Apnea prevalence among final anchors	39.01%

The final target-anchor set was identical across all context lengths ($k = 1, 3, 5, 7$, and 9), with the shorter-context tensors obtained as exact centered subsets of the corresponding $k = 9$ tensors. Invalid individual windows and rejected candidate anchors represent different counting units because a single invalid 60-s window may occur within the nine-epoch context of multiple candidate anchors. Recording-edge exclusions are likewise counted at the annotation level before candidate-anchor construction. Accordingly, these counts should not be summed as mutually exclusive components of a single exclusion total.

Participant-Grouped Cross-Validation Protocol

To prevent recordings from the same participant from appearing in different data partitions, participant-level grouping was performed before cross-validation fold construction. According to the participant information accompanying the Apnea-ECG database, recording c05 is associated with the same participant as x33, whereas c06 is listed separately. However, PhysioNet additionally documents that c05 and c06 originate from the same original recording, with c05 beginning 80 s later than c06, and notes that c06 may represent a corrected version of c05. Accordingly, c05 was excluded as a duplicate recording and c06 was retained for analysis. To preserve participant independence conservatively, c06 was assigned to the same participant group as x33 (P22), thereby propagating the participant association reported for c05 to the retained version of the recording. The remaining 69 recordings were consequently organized into 31 participant groups, and all recordings assigned to the same participant group were treated as an indivisible unit during fold construction. The complete participant-to-record mapping and corresponding outer-fold assignment are provided in Table S2.

The five outer folds were generated using a fixed split seed of 42. A total of 100,000 candidate participant-level assignments were evaluated, and the final assignment was selected using a composite balancing objective that considered the numbers of recordings, normal anchors, apneic anchors, total anchors, and apnea prevalence across folds.

The resulting fold-level distributions are summarized in Table S3. For each cross-validation run, one outer fold served as the test set, the next fold in cyclic order served as the validation set, and the remaining three folds were used for training. Specifically, test folds 0–4 were paired with validation folds 1, 2, 3, 4, and 0, respectively. The same fold assignment was retained for every context-length condition, apnea-label retention level, and experimental repetition.

Table S2: Participant-level grouping and outer-fold assignment.

Fold	Participant	Recordings	Normal	Apneic	Total	Apnea (%)	Mean AHI (events/h)
0	P02	a02, x14	151	851	1002	84.93	74.50
0	P10	a11	240	218	458	47.60	14.00
0	P11	a15, x27, x28	203	1274	1477	86.26	67.33
0	P14	b01, x03	904	31	935	3.32	0.18
0	P17	b05, x11	806	66	872	7.57	5.00
0	P23	c07, x34	878	8	886	0.90	0.19
0	P24	c10, x18	850	3	853	0.35	0.00
1	P08	a08, a13, x20	783	683	1466	46.59	42.33
1	P12	a17, x12	780	215	995	21.61	33.00
1	P19	c02, c09	929	3	932	0.32	0.00
1	P22	c06, x33	920	3	923	0.33	0.19
1	P29	x13, x26	379	630	1009	62.44	16.90
1	P31	x31, x32	146	933	1079	86.47	82.65
2	P01	a01, a14	133	849	982	86.46	62.15
2	P04	a04, a12	74	979	1053	92.97	78.80
2	P06	a06, x15	587	404	991	40.77	20.30
2	P15	b02, b03, x16, x21	1602	346	1948	17.76	21.50
2	P26	x06, x24	844	1	845	0.12	0.00
2	P30	x17, x22	853	3	856	0.35	0.00
3	P07	a07, a16, x01, x30	659	1333	1992	66.92	52.00
3	P13	a19, x05, x08, x25	882	1118	2000	55.90	41.00
3	P18	c01, x35	931	0	931	0.00	0.00
3	P21	c04, x29	924	0	924	0.00	0.00
3	P25	x02	256	205	461	44.47	37.70
3	P28	x10	405	96	501	19.16	10.00
4	P03	a03, x19	345	645	990	65.15	47.65
4	P05	a05, a10, a20, x07	1036	919	1955	47.01	31.00
4	P09	a09, a18	157	811	968	83.78	57.05
4	P16	b04, c08	908	10	918	1.09	0.35
4	P20	c03, x04	911	0	911	0.00	0.00
4	P27	x09, x23	732	286	1018	28.09	16.40

AHI values were obtained from the additional information accompanying the PhysioNet Apnea-ECG Database. Because several participant groups contained multiple recordings with different recording-level AHI values, Mean AHI denotes the arithmetic mean of the available AHI values across all recordings assigned to each participant group.

Table S3: Participant and class balance across the five outer cross-validation folds.

Fold	Participants	Records	Normal	Apneic	Total	Apnea (%)	AHI, Mean ± SD
0	7	14	4032	2451	6483	37.81	23.03 ± 33.15
1	6	13	3937	2467	6404	38.52	29.18 ± 31.27
2	6	14	4093	2582	6675	38.68	30.46 ± 32.80
3	6	14	4057	2752	6809	40.42	23.45 ± 22.83
4	6	14	4089	2671	6760	39.51	25.41 ± 24.01
Overall	31	69	20208	12923	33131	39.01	26.20 ± 27.47

AHI values are expressed as events/h. For participant groups containing multiple recordings, a participant-level AHI was first calculated as the mean of the recording-level AHI values reported in the original Apnea-ECG metadata. Fold-level values represent the mean ± standard deviation of these participant-level AHI values.

Multi-Window Context Configuration and Apnea-Label Retention Protocol

Apnea-label retention was performed independently within each training partition while leaving all normal training anchors unchanged. For each fold, only the specified proportion of real apnea

target anchors was retained, and the remaining apnea target samples were replaced by offline-augmented samples so that the effective positive-class sample count matched the corresponding full-retention condition. Thus, the retention ratio r refers specifically to real apnea target labels rather than to the complete training-label set. Exact fold-specific retention and augmentation counts are summarized in Table S4.

Three paired repetitions were generated using subsampling seeds 42, 43, and 44 and training seeds 142, 143, and 144, respectively. The number of retained apnea anchors was identical across repetitions for a given fold and retention ratio, although the identities of the retained anchors differed according to the repetition-specific subsampling seed. Within each repetition, the same retained-anchor set was used for all context lengths. Validation and test partitions were not subjected to label retention. An apnea epoch omitted as a supervised target was not removed from the underlying continuous ECG and could therefore remain present as an unlabeled neighboring context epoch when it fell within the temporal context of another retained target anchor.

Table S4: Fold-specific real apnea-label retention and offline augmentation counts used in the training partitions.

Fold	r (%)	Real Normal	Full Real Apnea	Retained Real Apnea	Augmented Apnea	Unique Real Total	Unique Real Labels (%)
0	5	12,239	8,005	400	7,605	12,639	62.43
0	10	12,239	8,005	801	7,204	13,040	64.41
0	20	12,239	8,005	1,601	6,404	13,840	68.37
0	40	12,239	8,005	3,202	4,803	15,441	76.27
0	60	12,239	8,005	4,803	3,202	17,042	84.18
0	80	12,239	8,005	6,404	1,601	18,643	92.09
0	100	12,239	8,005	8,005	0	20,244	100.00
1	5	12,178	7,874	394	7,480	12,572	62.70
1	10	12,178	7,874	787	7,087	12,965	64.66
1	20	12,178	7,874	1,575	6,299	13,753	68.59
1	40	12,178	7,874	3,150	4,724	15,328	76.44
1	60	12,178	7,874	4,724	3,150	16,902	84.29
1	80	12,178	7,874	6,299	1,575	18,477	92.15
1	100	12,178	7,874	7,874	0	20,052	100.00
2	5	12,058	7,589	379	7,210	12,437	63.30
2	10	12,058	7,589	759	6,830	12,817	65.24
2	20	12,058	7,589	1,518	6,071	13,576	69.10
2	40	12,058	7,589	3,036	4,553	15,094	76.83
2	60	12,058	7,589	4,553	3,036	16,611	84.55
2	80	12,058	7,589	6,071	1,518	18,129	92.27
2	100	12,058	7,589	7,589	0	19,647	100.00
3	5	12,062	7,500	375	7,125	12,437	63.58
3	10	12,062	7,500	750	6,750	12,812	65.49
3	20	12,062	7,500	1,500	6,000	13,562	69.33
3	40	12,062	7,500	3,000	4,500	15,062	77.00
3	60	12,062	7,500	4,500	3,000	16,562	84.66
3	80	12,062	7,500	6,000	1,500	18,062	92.33
3	100	12,062	7,500	7,500	0	19,562	100.00
4	5	12,087	7,801	390	7,411	12,477	62.74
4	10	12,087	7,801	780	7,021	12,867	64.70
4	20	12,087	7,801	1,560	6,241	13,647	68.62
4	40	12,087	7,801	3,120	4,681	15,207	76.46
4	60	12,087	7,801	4,681	3,120	16,768	84.31
4	80	12,087	7,801	6,241	1,560	18,328	92.16
4	100	12,087	7,801	7,801	0	19,888	100.00

"Unique Real Total" denotes the number of distinct real training target anchors remaining after apnea-label retention and includes all normal anchors plus the retained real apnea anchors. "Unique Real Labels (%)" is this quantity divided by the corresponding full real training-set size. For each fold and retention level, the sum of retained real apnea and augmented apnea samples equals the full real apnea count, so the effective training-set size remained equal to that of the $r = 100\%$ condition. The counts were identical across the three repetitions, although different apnea-anchor identities were selected using the repetition-specific subsampling seeds.

Dual-Stage Augmentation Strategy

Offline augmentation was used only to generate additional apnea-target training samples when $r < 100\%$. For each required synthetic sample, one retained real apnea-centered multi-window block was randomly selected with replacement from the available real apnea training pool. Each constituent 60-s window of the selected block was then augmented independently, such that transformation occurrence and magnitude were sampled separately for each window rather than

shared across the complete multi-window block. Augmentation types, transformation probabilities, and magnitude ranges were kept consistent across all context-length and apnea-label retention conditions whenever augmentation was applied; only the number of offline-generated apnea samples varied according to r.

Online augmentation was applied whenever a training sample was retrieved, irrespective of whether the sample was a real normal anchor, a retained real apnea anchor, or an offline-generated apnea sample. Consequently, offline-generated apnea samples first received the offline transformations during dataset expansion and remained eligible for additional stochastic online perturbation during training. Online transformations were independently sampled for each constituent 60-s window. No augmentation was applied to the validation or test partitions. The complete augmentation settings are summarized in Table S5.

Table S5: Offline and online augmentation settings applied to each constituent 60-s ECG window.

Stage	Transformation	Probability	Magnitude / Implementation
Offline	Temporal shift	0.70	Circular shift within ±2% of the window length
Offline	Amplitude scaling	0.70	Multiplicative factor sampled from $[0.95, 1.05]$
Offline	Gaussian noise	0.70	Noise standard deviation equal to 0.5%–1.5% of the window standard deviation
Offline	Sinusoidal baseline perturbation	0.50	Amplitude equal to 1%–3% of the window standard deviation
Offline	Short dropout	0.40	One contiguous interval spanning 0.5%–1.0% of the window set to zero
Offline	Light time warping	0.50	Temporal scaling factor sampled from $[0.99, 1.01]$, with the output restored to the original length
Online	Temporal shift	0.50	Circular shift within ±3% of the window length
Online	Amplitude jitter	0.50	Multiplicative factor sampled from $[0.90, 1.10]$
Online	Gaussian noise	0.50	Noise standard deviation equal to 2% of the window standard deviation
Online	Short time mask	0.30	One contiguous interval spanning 1%–3% of the window set to zero

All transformation decisions and associated random parameters were sampled independently for each constituent window within a multi-window block. Offline transformations were used only for synthetic apnea-target generation, whereas online transformations were applied to every training sample. Validation and test samples were evaluated without augmentation.

Attention-Pooled CNN-BiLSTM Architecture

The network architecture is described in the main manuscript. Complete optimization, training, sampling, EMA, and model-selection settings are summarized in Table S6. The EMA parameter set was used for validation and test inference at each epoch. For the primary analysis, each experimental run was represented by the epoch yielding the highest validation F1-score. The classification threshold was determined exclusively from validation predictions and was subsequently applied unchanged to the corresponding test predictions. Test-set performance was not used for primary checkpoint or threshold selection.

Table S6: Complete training, optimization, and model-selection settings.

Setting	Implementation
Optimizer	AdamW
Initial learning rate	1×10^{-3}
Weight decay, backbone	5×10^{-4}
Weight decay, output head	0
Training epochs	80
Batch size	64
Learning-rate warmup	4 epochs (5% of total training)
Post-warmup schedule	Cosine decay
Gradient clipping	Maximum gradient norm of 1.0
Loss function	BCEWithLogitsLoss
Positive-class weighting	Ratio of effective normal to apnea training samples within each run
Training sampler	WeightedRandomSampler with inverse class-frequency weights
Sampler replacement	Yes
Samples drawn per epoch	Equal to the effective training-set size
Output-head dropout	0.3
EMA decay	0.999
EMA use	Validation and test inference at every epoch and saved primary checkpoint
CNN projection dimension (d_{model})	192
BiLSTM hidden dimension	192 per direction
BiLSTM layers	1
BiLSTM directionality	Bidirectional
Attention pooling	Trainable scalar attention score followed by softmax over the complete kT sequence
Primary checkpoint selection	Epoch with maximum validation F1-score
Threshold candidate range	0.10–0.90
Threshold increment	0.01
Number of threshold candidates	81
Threshold selection criterion	Maximum validation F1-score
Threshold smoothing	Mean of the validation-selected thresholds from up to the five most recent epochs
Primary test threshold	Smoothed validation-derived threshold applied unchanged
Fixed-threshold robustness analysis	Threshold = 0.50
Test-set information used for primary selection	No
Training DataLoader workers	4
Validation/test DataLoader workers	4
Persistent DataLoader workers	No

Supplementary Results

Representative Confusion Matrices

To provide a class-specific view of the classification behavior, Figure S1 presents representative confusion matrices comparing the single-window baseline with the best-performing multi-window configuration at selected apnea-label retention levels. The selected conditions include the two most label-restricted settings ($r = 5\%$ and 10%) and the full-retention setting ($r = 100\%$). For each value of r, the $k = 1$ condition is compared with the multi-window configuration yielding the highest mean F1-score ($k = 7$, 9, and 5 for $r = 5\%$, 10%, and 100%, respectively).

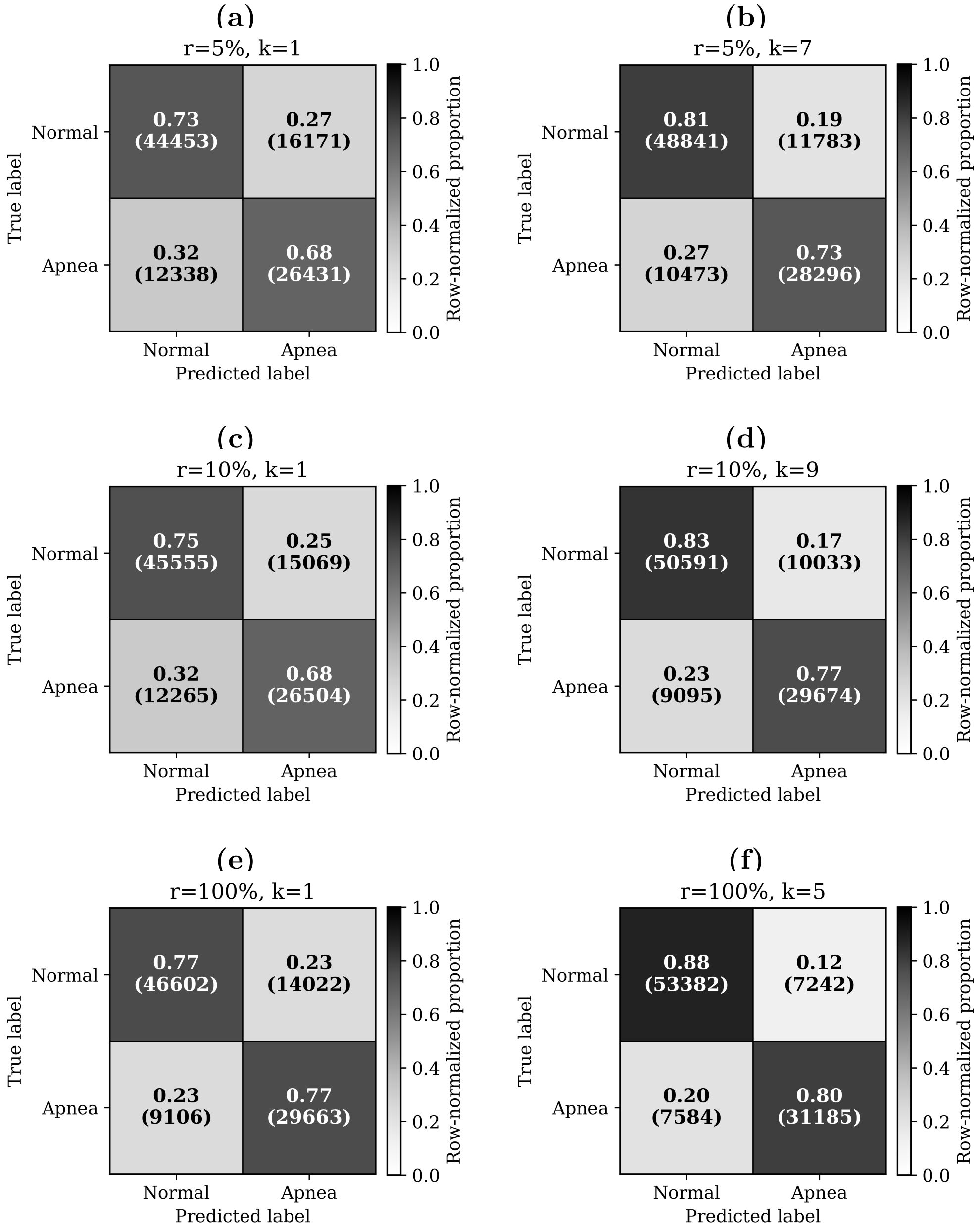

Figure S1: Representative aggregated confusion matrices for selected single-window and multi-window conditions. (a) $r = 5\%$, $k = 1$; (b) $r = 5\%$, $k = 7$; (c) $r = 10\%$, $k = 1$; (d) $r = 10\%$, $k = 9$; (e) $r = 100\%$, $k = 1$; and (f) $r = 100\%$, $k = 5$. Each matrix aggregates test predictions across three training repetitions and five participant-grouped folds ($n = 15$ runs per condition). Cell values show the row-normalized proportion followed by the aggregated prediction count in parentheses. All panels use the same grayscale range from 0 to 1.

Each confusion matrix was obtained by aggregating test-set predictions across three training repetitions and five participant-grouped folds ($n = 15$ test runs per condition). Rows correspond to the true class and columns to the predicted class. The first value in each cell represents the row-normalized proportion, whereas the value in parentheses represents the aggregated number of prediction instances. Because each participant-grouped test fold was evaluated once in each of the three training repetitions, the displayed counts represent aggregated prediction instances rather than unique target anchors. For every run, predictions were obtained from the checkpoint selected by maximum validation F1-score using the corresponding validation-derived classification threshold.

Representative Training Dynamics

To provide additional insight into the training process, Figure S2 presents representative training and validation loss trajectories for the best-performing multi-window configurations at $r = 5\%$, 10%, and 100%. These conditions correspond to $k = 7$, 9, and 5, respectively. For each condition, the trajectories were summarized across three training repetitions and five participant-grouped folds ($n = 15$ runs). The solid curves represent the mean loss, and the shaded regions indicate ±1 standard deviation across runs.

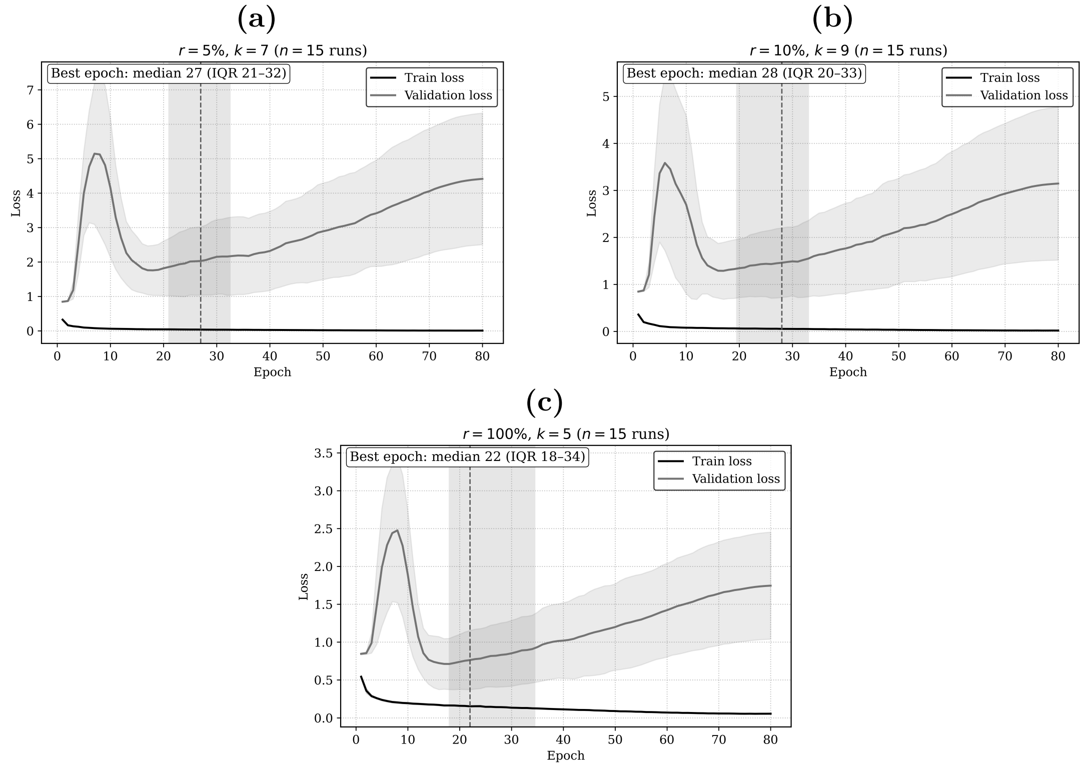

Figure S2: Representative training dynamics for selected multi-window configurations. Panels (a)–(c) correspond to the representative conditions $r = 5\%, k = 7$, $r = 10\%, k = 9$, and $r = 100\%, k = 5$, respectively. For each condition, the trajectories are summarized across three training repetitions and five participant-grouped folds ($n = 15$ runs). The solid curves represent the mean loss, and the shaded regions indicate ±1 standard deviation across runs. The vertical dashed line indicates the median epoch of the checkpoint selected by maximum validation F1-score across the 15 runs, and the gray vertical band indicates the corresponding interquartile range.

Training loss represents the mean optimization loss calculated from augmented mini-batches drawn using inverse class-frequency weighted sampling, with stochastic dropout active and the non-EMA model parameters. In contrast, validation loss was calculated on the unaugmented validation partition using the EMA model. Accordingly, the absolute magnitudes and temporal trajectories of training and validation loss are not expected to coincide directly.

The vertical dashed line indicates the median epoch of the checkpoint selected by maximum validation F1-score across the 15 runs, and the vertical shaded band indicates the corresponding interquartile range. Because binary cross-entropy loss is sensitive to prediction confidence and calibration, whereas F1-score depends on thresholded class predictions, the epoch yielding the highest validation F1-score does not necessarily coincide with the epoch yielding the minimum validation loss. The relatively low validation loss during the earliest epochs should therefore not be interpreted as superior initial classification performance. Similarly, the later tendency for training loss to continue decreasing while validation loss increases is consistent with increasing overfitting during prolonged training and further supports the use of validation-based checkpoint

selection rather than the final training epoch.

Representative Mean-Beat Morphology under Offline Augmentation

To provide a direct visual assessment of ECG morphology under the present augmentation protocol, representative mean-beat waveforms were compared for the two most restrictive apnea-label retention conditions (r = 5% and 10%). For each condition, R-peak-aligned beats were pooled across the three experimental repetitions and five participant-grouped folds, with equal contribution from each fold–repetition run. The mixed condition reflects the effective apnea-class training composition at the corresponding retention level, comprising retained real apnea samples and offline-augmented variants in their respective training proportions. The shaded region represents the variability of the retained real apnea beats, while the solid and dashed curves represent the corresponding real and mixed mean waveforms.

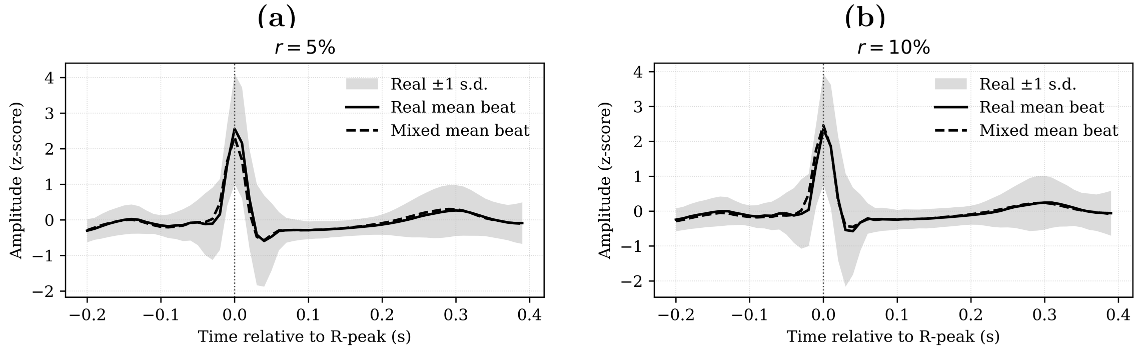

Figure S3: Representative pooled mean-beat morphology under low apnea-label retention. (a) $r = 5\%$ and (b) $r = 10\%$. Solid curves indicate the mean waveform of retained real apnea beats, dashed curves indicate the mean waveform sampled from the effective mixed apnea training pool (retained real plus offline-augmented samples), and gray bands indicate ± 1 standard deviation of the retained real beats. Beats were aligned relative to the detected R-peak and pooled with equal contribution from each of the three training repetitions and five participant-grouped folds. The comparison provides a qualitative assessment of whether the applied offline perturbations introduce gross alterations in beat morphology under conditions with the greatest reliance on augmented apnea samples.

Performance Dynamics across Apnea-Label Retention Levels

Complete descriptive performance across all 35 experimental conditions is provided in Table S7. Values represent means and standard deviations across three training repetitions and five participant-grouped folds (n = 15 runs per condition).

Table S7: Complete test-set performance across the 35 combinations of apnea-label retention ratio and context length.

r (%)	k	**F1 (%)**	**AUPRC (%)**	**AUROC (%)**	**Accuracy (%)**
5	1	64.66 ± 5.55	68.58 ± 8.81	76.04 ± 6.68	71.36 ± 5.78
5	3	70.28 ± 6.63	69.19 ± 11.92	79.91 ± 6.28	76.21 ± 5.82
5	5	70.97 ± 5.12	68.83 ± 14.33	80.79 ± 7.40	76.91 ± 5.64
5	7	71.84 ± 5.97	70.49 ± 15.64	80.94 ± 10.53	77.52 ± 7.58
5	9	71.30 ± 7.03	68.82 ± 14.03	81.17 ± 7.66	77.47 ± 7.26
10	1	65.48 ± 8.09	69.24 ± 10.12	75.88 ± 8.75	72.42 ± 6.95
10	3	72.36 ± 4.31	71.10 ± 12.20	82.36 ± 5.24	77.88 ± 3.84
10	5	74.17 ± 7.76	73.24 ± 12.93	84.79 ± 5.94	80.15 ± 5.11
10	7	74.64 ± 6.16	72.36 ± 14.22	84.41 ± 7.26	80.44 ± 4.22
10	9	75.50 ± 3.56	74.16 ± 11.76	85.81 ± 4.24	80.73 ± 3.12
20	1	66.64 ± 6.50	71.54 ± 8.37	78.93 ± 6.07	73.38 ± 5.17
20	3	74.94 ± 4.63	75.88 ± 10.19	85.85 ± 4.26	79.71 ± 4.14
20	5	76.78 ± 4.75	76.61 ± 12.03	86.94 ± 5.06	81.87 ± 3.43
20	7	74.47 ± 7.19	74.06 ± 14.35	85.43 ± 7.03	79.97 ± 5.66
20	9	77.60 ± 5.18	79.37 ± 12.56	88.38 ± 5.55	82.57 ± 4.25
40	1	66.09 ± 10.76	72.29 ± 10.71	79.65 ± 6.96	72.42 ± 6.34
40	3	76.42 ± 4.04	79.43 ± 10.52	87.69 ± 4.36	80.85 ± 3.06
40	5	78.32 ± 4.27	83.46 ± 8.15	89.44 ± 3.62	82.80 ± 2.98
40	7	77.91 ± 4.06	81.39 ± 8.84	89.04 ± 3.71	82.56 ± 2.82
40	9	78.26 ± 4.58	81.79 ± 7.61	89.42 ± 3.84	83.14 ± 3.01
60	1	70.10 ± 8.18	73.22 ± 10.40	80.33 ± 7.61	74.30 ± 8.80
60	3	77.71 ± 3.94	81.79 ± 8.65	89.35 ± 3.27	81.66 ± 2.43
60	5	78.39 ± 4.76	81.32 ± 12.86	88.88 ± 5.34	82.92 ± 3.25
60	7	77.00 ± 6.39	80.46 ± 8.62	89.15 ± 3.56	81.94 ± 3.62
60	9	78.34 ± 5.33	82.99 ± 10.63	90.15 ± 4.32	83.06 ± 3.17
80	1	71.07 ± 7.95	77.58 ± 7.75	83.26 ± 7.36	75.83 ± 7.51
80	3	78.80 ± 5.02	87.67 ± 4.28	90.72 ± 3.83	83.33 ± 3.82
80	5	79.35 ± 4.48	89.40 ± 3.01	92.00 ± 3.06	83.84 ± 3.11
80	7	78.36 ± 4.89	86.27 ± 7.81	89.85 ± 5.12	83.09 ± 3.11
80	9	78.35 ± 5.46	87.92 ± 3.60	90.78 ± 3.60	83.54 ± 3.25
100	1	71.48 ± 8.85	78.91 ± 9.28	83.31 ± 8.32	76.73 ± 6.17
100	3	78.96 ± 5.74	87.95 ± 5.21	90.64 ± 4.45	83.54 ± 4.57
100	5	80.77 ± 5.10	89.85 ± 4.05	92.54 ± 3.09	85.09 ± 4.39
100	7	78.85 ± 4.76	88.31 ± 2.81	91.14 ± 2.69	83.71 ± 3.62
100	9	78.88 ± 3.96	89.04 ± 2.11	91.71 ± 1.73	84.37 ± 1.76

Values are means ± SD across three training repetitions and five participant-grouped folds ($n = 15$ runs per condition). All metrics were obtained from the checkpoint selected by maximum validation F1-score using the corresponding validation-derived classification threshold.

Statistical Effects of Context Length and Apnea-Label Retention

Planned paired comparisons were performed between each multi-window configuration ($k = 3, 5, 7, 9$) and the corresponding single-window baseline ($k = 1$) at each apnea-label retention level. Table S8 reports the mean paired difference in test-set F1-score, the 95% confidence interval obtained from the two-way crossed bootstrap, the number of participant-grouped folds showing a positive mean difference after averaging across training repetitions, and the exact one-sided sign-flip sensitivity results.

Table S8: Planned F1-score comparisons between multi-window configurations and the corresponding single-window baseline.

r (%)	k	**ΔF1 (pp)**	**Crossed-Bootstrap 95% CI**	**Positive Folds**	**Exact p**	**Holm-Adjusted p**
5	3	+5.62	[0.56, 12.30]	4/5	0.06250	0.875
5	5	+6.31	[0.39, 12.21]	4/5	0.09375	0.875
5	7	+7.18	[0.70, 12.91]	4/5	0.06250	0.875
5	9	+6.64	[-2.66, 14.45]	3/5	0.12500	0.875
10	3	+6.88	[1.99, 11.51]	5/5	0.03125	0.875
10	5	+8.69	[2.32, 14.60]	5/5	0.03125	0.875
10	7	+9.16	[4.07, 14.59]	5/5	0.03125	0.875
10	9	+10.02	[5.10, 15.33]	5/5	0.03125	0.875
20	3	+8.30	[3.35, 12.82]	5/5	0.03125	0.875
20	5	+10.14	[6.35, 14.32]	5/5	0.03125	0.875
20	7	+7.83	[2.48, 12.97]	5/5	0.03125	0.875
20	9	+10.96	[5.64, 16.07]	5/5	0.03125	0.875
40	3	+10.34	[4.35, 19.64]	5/5	0.03125	0.875
40	5	+12.23	[4.92, 22.21]	5/5	0.03125	0.875
40	7	+11.82	[3.61, 21.80]	5/5	0.03125	0.875
40	9	+12.17	[4.24, 22.11]	5/5	0.03125	0.875
60	3	+7.62	[2.03, 14.13]	5/5	0.03125	0.875
60	5	+8.29	[1.28, 16.43]	4/5	0.06250	0.875
60	7	+6.90	[1.46, 14.77]	5/5	0.03125	0.875
60	9	+8.25	[2.33, 16.55]	4/5	0.06250	0.875
80	3	+7.74	[3.99, 11.33]	5/5	0.03125	0.875
80	5	+8.28	[4.06, 13.29]	5/5	0.03125	0.875
80	7	+7.29	[2.85, 12.54]	5/5	0.03125	0.875
80	9	+7.29	[1.06, 13.14]	4/5	0.06250	0.875
100	3	+7.48	[4.10, 10.87]	5/5	0.03125	0.875
100	5	+9.29	[4.77, 14.06]	5/5	0.03125	0.875
100	7	+7.38	[2.44, 12.29]	5/5	0.03125	0.875
100	9	+7.40	[1.46, 14.02]	4/5	0.06250	0.875

ΔF1 denotes the paired mean difference between the indicated multi-window configuration and the corresponding $k = 1$ condition. Confidence intervals were obtained from 10,000 two-way crossed bootstrap resamples of training repetitions and participant-grouped folds. "Positive Folds" indicates the number of the five participant-grouped folds in which the mean difference remained positive after averaging across the three training repetitions. Exact one-sided sign-flip tests were performed on these five fold-level differences, and the resulting 28 p-values were adjusted using the Holm procedure.

Supplementary Methodological Analysis

Apnea-Related Exposure through Unlabeled Neighboring Context

Because label retention affected only supervised central apnea targets, apnea epochs excluded from the supervised target set could remain present as unlabeled neighboring context for other retained anchors. To quantify this effect, neighboring-window composition was evaluated at the two lowest apnea-label retention levels ($r = 5\%$ and 10%) across all multi-window configurations. Values in Table S9 are reported as the mean ± standard deviation across three paired repetitions and five participant-grouped folds.

Table S9: Apnea-related exposure through unlabeled neighboring context under low apnea-label retention.

r	k	Apnea neighbors (%)	Blocks with apnea neighbor (%)	Removed apnea re-exposed (%)
5%	3	5.86 ± 0.18	8.53 ± 0.30	17.37 ± 0.33
5%	5	6.79 ± 0.24	12.54 ± 0.47	30.52 ± 0.62
5%	7	7.54 ± 0.29	15.95 ± 0.63	41.14 ± 0.83
5%	9	8.20 ± 0.33	18.91 ± 0.77	49.93 ± 0.97
10%	3	8.54 ± 0.22	11.24 ± 0.33	25.81 ± 0.43
10%	5	9.39 ± 0.27	15.14 ± 0.49	44.04 ± 0.84
10%	7	10.08 ± 0.32	18.45 ± 0.64	57.47 ± 1.16
10%	9	10.69 ± 0.36	21.32 ± 0.77	67.49 ± 1.30

"Apnea neighbors" denotes the proportion of neighboring window occurrences whose original reference annotation was apnea. "Blocks with apnea neighbor" denotes the proportion of supervised target blocks containing at least one apnea-labeled neighboring epoch. "Removed apnea re-exposed" denotes the proportion of apnea epochs excluded from the supervised target set that nevertheless appeared at least once as an unlabeled neighboring window. Neighboring reference labels were used only for this post hoc descriptive analysis and were never provided to the model or used in the training loss.

Supplementary Robustness Analysis

Fixed-Threshold Performance

To evaluate whether the multi-window performance pattern depended on validation-derived classification thresholds, the EMA checkpoint selected using validation F1 in each run was additionally evaluated on the corresponding test partition using a fixed threshold of 0.5. No test-set information was used for checkpoint selection or threshold determination. Table S10 summarizes performance across all 35 experimental conditions in the fixed-threshold robustness analysis.

Table S10: Test-set performance in the fixed-threshold robustness analysis.

r (%)	k	**F1 (%)**	**AUPRC (%)**	**AUROC (%)**	**Accuracy (%)**
5	1	62.43 ± 5.73	68.58 ± 8.81	76.04 ± 6.68	72.27 ± 5.56
5	3	66.00 ± 7.77	69.19 ± 11.92	79.91 ± 6.28	75.70 ± 5.42
5	5	64.46 ± 7.65	68.83 ± 14.33	80.79 ± 7.40	75.52 ± 6.04
5	7	64.30 ± 8.61	70.49 ± 15.64	80.94 ± 10.53	75.21 ± 8.20
5	9	63.52 ± 5.99	68.82 ± 14.03	81.17 ± 7.66	74.50 ± 6.38
10	1	63.99 ± 8.82	69.24 ± 10.12	75.88 ± 8.75	73.73 ± 6.53
10	3	69.85 ± 5.83	71.10 ± 12.20	82.36 ± 5.24	78.28 ± 3.99
10	5	69.01 ± 10.82	73.24 ± 12.93	84.79 ± 5.94	78.77 ± 6.45
10	7	68.40 ± 9.68	72.36 ± 14.22	84.41 ± 7.26	78.46 ± 6.08
10	9	69.39 ± 5.42	74.16 ± 11.76	85.81 ± 4.24	79.11 ± 3.93
20	1	65.33 ± 9.56	71.54 ± 8.37	78.93 ± 6.07	75.04 ± 4.39
20	3	72.12 ± 7.48	75.88 ± 10.19	85.85 ± 4.26	80.32 ± 4.22
20	5	72.70 ± 6.50	76.61 ± 12.03	86.94 ± 5.06	80.99 ± 4.35
20	7	70.02 ± 10.37	74.06 ± 14.35	85.43 ± 7.03	79.12 ± 6.86
20	9	72.90 ± 8.56	79.37 ± 12.56	88.38 ± 5.55	81.47 ± 5.48
40	1	64.67 ± 11.72	72.29 ± 10.71	79.65 ± 6.96	74.42 ± 6.18
40	3	75.19 ± 6.18	79.43 ± 10.52	87.69 ± 4.36	81.79 ± 4.01
40	5	76.96 ± 5.78	83.46 ± 8.15	89.44 ± 3.62	83.53 ± 3.50
40	7	74.88 ± 6.13	81.39 ± 8.84	89.04 ± 3.71	82.28 ± 3.78
40	9	74.74 ± 6.83	81.79 ± 7.61	89.42 ± 3.84	82.50 ± 3.79
60	1	68.63 ± 8.17	73.22 ± 10.40	80.33 ± 7.61	75.39 ± 8.30
60	3	77.82 ± 4.49	81.79 ± 8.65	89.35 ± 3.27	82.96 ± 2.66
60	5	77.95 ± 5.62	81.32 ± 12.86	88.88 ± 5.34	83.35 ± 3.83
60	7	76.80 ± 6.35	80.46 ± 8.62	89.15 ± 3.56	82.72 ± 3.74
60	9	77.48 ± 6.44	82.99 ± 10.63	90.15 ± 4.32	83.49 ± 3.68
80	1	71.01 ± 8.72	77.58 ± 7.75	83.26 ± 7.36	77.07 ± 8.78
80	3	78.78 ± 4.50	87.67 ± 4.28	90.72 ± 3.83	84.06 ± 3.18
80	5	79.79 ± 3.74	89.40 ± 3.01	92.00 ± 3.06	84.76 ± 2.28
80	7	78.97 ± 4.47	86.27 ± 7.81	89.85 ± 5.12	84.03 ± 2.90
80	9	77.63 ± 6.55	87.92 ± 3.60	90.78 ± 3.60	83.56 ± 3.17
100	1	71.00 ± 9.47	78.91 ± 9.28	83.31 ± 8.32	78.45 ± 5.96
100	3	78.29 ± 5.82	87.95 ± 5.21	90.64 ± 4.45	84.22 ± 3.70
100	5	80.11 ± 4.12	89.85 ± 4.05	92.54 ± 3.09	85.34 ± 3.20
100	7	78.05 ± 3.65	88.31 ± 2.81	91.14 ± 2.69	83.87 ± 2.18
100	9	78.89 ± 3.37	89.04 ± 2.11	91.71 ± 1.73	84.74 ± 1.57

Values are means ± SD across three training repetitions and five participant-grouped folds ($n = 15$ runs per condition). The same validation-selected EMA checkpoints used in the primary analysis were evaluated using a fixed classification threshold of 0.5. AUPRC and AUROC are threshold-independent and therefore identical to the corresponding values in Supplementary Table S7.
